# Outlining cardiac ion channel protein interactors and their signature in the human electrocardiogram

**DOI:** 10.1038/s44161-023-00294-y

**Published:** 2023-07-13

**Authors:** Svetlana Maurya, Robert W. Mills, Konstantin Kahnert, David Y. Chiang, Giorgia Bertoli, Pia R. Lundegaard, Marta Perez-Hernandez Duran, Mingliang Zhang, Eli Rothenberg, Alfred L. George, Calum A. MacRae, Mario Delmar, Alicia Lundby

**Affiliations:** 1 Department of Biomedical Sciences, Faculty of Health and Medical Sciences, University of Copenhagen, Copenhagen, Denmark; 2 Cardiovascular Medicine Division, Brigham and Women’s Hospital and Harvard Medical School, Boston, MA USA; 3 Division of Cardiology, NYU School of Medicine, New York, NY USA; 4 Division of Pharmacology, NYU School of Medicine, New York, NY USA; 5 Department of Pharmacology, Northwestern University Feinberg School of Medicine, Chicago, IL USA; 6 The Novo Nordisk Foundation Center for Protein Research, Faculty of Health and Medical Sciences, University of Copenhagen, Copenhagen, Denmark

**Keywords:** Protein analysis, Proteomics, Data integration, Cardiovascular biology

## Abstract

Protein–protein interactions are essential for normal cellular processes and signaling events. Defining these interaction networks is therefore crucial for understanding complex cellular functions and interpretation of disease-associated gene variants. We need to build a comprehensive picture of the interactions, their affinities and interdependencies in the specific organ to decipher hitherto poorly understood signaling mechanisms through ion channels. Here we report the experimental identification of the ensemble of protein interactors for 13 types of ion channels in murine cardiac tissue. Of these, we validated the functional importance of ten interactors on cardiac electrophysiology through genetic knockouts in zebrafish, gene silencing in mice, super-resolution microscopy and patch clamp experiments. Furthermore, we establish a computational framework to reconstruct human cardiomyocyte ion channel networks from deep proteome mapping of human heart tissue and human heart single-cell gene expression data. Finally, we integrate the ion channel interactome with human population genetics data to identify proteins that influence the electrocardiogram (ECG). We demonstrate that the combined channel network is enriched for proteins influencing the ECG, with 44% of the network proteins significantly associated with an ECG phenotype. Altogether, we define interactomes of 13 major cardiac ion channels, contextualize their relevance to human electrophysiology and validate functional roles of ten interactors, including two regulators of the sodium current (epsin-2 and gelsolin). Overall, our data provide a roadmap for our understanding of the molecular machinery that regulates cardiac electrophysiology.

## Abstract (web summary)

Maurya et al. define interactomes of 13 major cardiac ion channels, contextualize their relevance to human electrophysiology by integrating multi-omics data that suggest that 44% of the network proteins influence human electrocardiogram, and validate the functional roles of ten interactors.

## Main

Proteins rarely operate as single entities, but rather as components of co-evolved functionally interdependent complexes. Ion channels are critical to the organized electrical and mechanical functions of the heart. The amplitude and time course of the electrical current passing through an ion channel can be modulated either directly by modifications of the pore-forming protein itself, or indirectly through regulation of its partners^1,2^ or microenvironment. Although modulation of channel gating by auxiliary subunits is broadly appreciated, the electrophysiologically relevant channel interactome extends further and can include proteins involved in proper channel expression and localization. For example, variants of the scaffolding adaptor protein, ankyrin-B, have been associated with various forms of arrhythmia^3^. Channels also perform nonconductive functions, such as regulating intracellular signaling pathways^4–6^, and for these functions yet other interaction partners are involved. Previous studies have found that the number of partners in an ion channel complex can reach into the hundreds^1,7,8^ and their interactions can be quite dynamic as well as relatively steady state^7,9,10^. It is reasonable to speculate that, while some molecules will interact directly with the pore-forming channel protein, others will do so indirectly, through a third party (or more). Channels also interact with one another forming macromolecular complexes to orchestrate the cardiac electrical impulse^11–13^. For most ion channels, however, the identity of the components of their respective molecular networks is largely unknown. Delineating the protein network architecture of cardiac ion channels emerges as a strategy to understand the molecular bases of heart rhythm. In addition, more nuanced disease-associated proteins can be found in the interaction network of proteins with a more immediate association with disease^9,14^. This suggests that additional proteins that contribute to cardiac electrophysiological properties are yet to be found in the protein interaction network of cardiac ion channels.

Integration of genomic and protein network datasets has successfully pinpointed biological functions involved in genetically driven disease phenotypes^7,14,15^. Genomics datasets, such as those obtained from genome-wide association studies (GWAS) or exome sequencing, are becoming increasingly available through resources such as the UK Biobank^16^. Data from cardiac genetics and cardiac interaction proteomics contain fairly orthogonal sets of information and leveraging the combination of the two can provide molecular insights. High-resolution proteomics technologies foster unbiased investigations of tissue-specific protein–protein interactions, for instance by immunoprecipitation (IP) followed by tandem mass spectrometry (MS)^7,17–19^. We and others have previously shown how integrating protein interaction with genetics data can contribute molecular insights to mechanisms of cardiac disease^7,14,20^.

In this article, we expand our previous efforts and present a comprehensive map of cardiac ion channel proteins and their network of interaction partners obtained experimentally from murine cardiac tissue. The strategy outlines protein interaction networks encompassing direct as well as indirect interactors. We unveil 13 different cardiac ion channel networks utilizing affinity purification and mass spectrometry (MS)-based proteomics. All 13 channels are essential for proper cardiac function. The identified channel networks provide a roadmap for an agnostic approach to studying regulators of cardiac ion channel function. To emphasize channel interactors likely to influence cardiac electrophysiology, we intersected the channel networks with protein expression and single-cell RNA sequencing (scRNA-seq) data from human hearts and integrated it with human genetics data from population-based analysis of the electrocardiogram (ECG). Finally, we performed functional studies using optical mapping, super-resolution imaging and electrophysiology to obtain a first look at the physiological relevance of several interactors identified using this network. The functional experiments illustrate the new information on channel interactors contained in the large channel network presented, and it presents epsin-2 and gelsolin as regulators of the sodium current in cardiomyocytes.

## Results

### Interaction networks of 13 cardiac ion channels

**Fig. 1:**
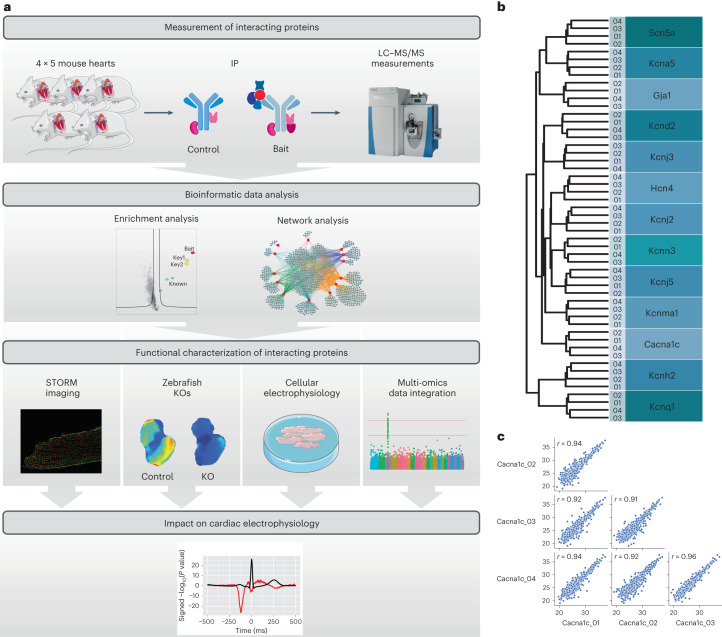
MS evaluation of cardiac ion channel IPs. **a**, Workflow of the study. We performed MS measurements of immunoprecipitated channels and their interactors and of control IPs from quadruplicate murine cardiac tissue lysates. Deep proteome measurements of the membrane-enriched mouse heart samples utilized in the IP experiments were also performed. Bioinformatics network analyses prioritized interactors for functional evaluation. A subset of interactors were evaluated for their functional impact on cardiac electrophysiology by STORM imaging, optical mapping in zebrafish KOs, and patch clamping of cardiomyocytes from mice with interactor genes silenced. From multi-omics data integration, the impact of each interactor in human electrophysiology is evaluated. **b**, Dendrogram from unsupervised hierarchical cluster analysis of protein intensities of proteins identified in IP experiments show that the four replicate experiments all cluster together. The clustering follows the bait replicates. **c**, Pearson correlation coefficients for protein intensities of the four Cacna1c replicate pulldown experiments. Pearson correlation coefficients are indicated in each scatter plot. Parts of the figure were drawn by using pictures from Servier Medical Art. Servier Medical Art by Servier is licensed under a Creative Commons Attribution 3.0 Unported License (https://creativecommons.org/licenses/by/3.0/). Source data

**Extended Data Fig. 1:**
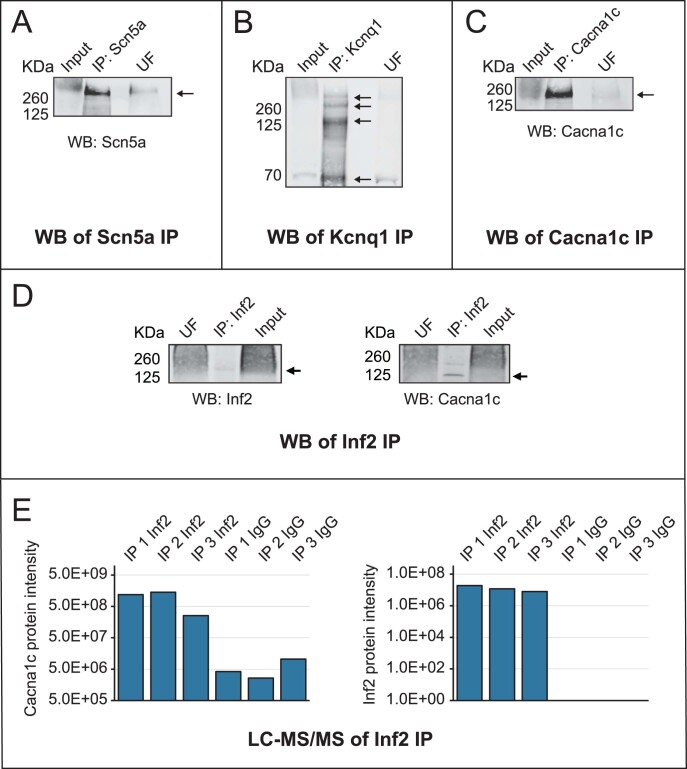
Immunoblot validation of bait immunoprecipitations. Immunoprecipitated channels Scn5a **a**), Kcnq1 **b**), and Cacna1c **c**) evaluated by western blot. **d**) Inf2 (Cacna1c interactor) was immunoprecipitated from murine cardiac tissue and evaluated by western blotting for the presence of Cacna1c (left panel, probed with IRDye 800CW secondary antibody) to confirm co-immunoprecipitation with Inf2. The same blot was evaluated for presence of Inf2 (right panel, probed with IRDye 680LT secondary antibody) to ensure that Inf2 was immunoprecipitated. In all the panels, black arrows denote the band of interest. The lowermost arrow in Panel B shows the Kcnq1 monomer band and the higher order oligomers are shown by the upper arrows. UF denotes unbound fraction of the immunoprecipitation (IP) experiment. The immunoblot validation was carried out thrice for panels A-C and twice for panel D with reproducible results. **e**) LC-MS/MS analysis of Inf2 IPs evaluating Cacna1c (left) and Inf2 protein abundances (right). Triplicate immunoprecipitations were performed from murine cardiac tissue using antibodies against Inf2 or IgG. Precipitated proteins were evaluated by mass spectrometry. Inf2 was abundantly present in the three Inf2 IPs and was absent in the triplicate IgG control Ips (right). The calcium channel protein Cacna1c was identified in all six immunoprecipitations but was a hundred-fold more abundant in the Inf2 IPs than in the control IPs (left). Source data

We carried out an initial screen of 40 antibodies targeting cardiac ion channels using MS-based proteomics. From this, we identified 13 antibodies to discrete channels with sufficient binding specificity for further studies (Table 1 and Supplementary Table 1). Evaluated antibodies were selected if they gave the channel bait among the most abundant proteins in affinity purification (AP)–MS/MS experiments. We immunoprecipitated the cognate 13 channels from membrane-enriched cardiac lysates of male mice in quadruplicate and analyzed the precipitated protein components using online reverse-phase liquid chromatography coupled to a Q-Exactive HF-X mass spectrometer (Fig. 1a). For concise and consistent representation of protein names for all protein interactors, we used the corresponding gene names without italics as the protein symbol. We performed additional pulldown experiments and characterized deep proteomes from the membrane-enriched mouse heart samples. Western blot and MS/MS confirmation for a subset of the bait and interacting proteins is shown in Extended Data Fig. 1.

**Table 1:** Summary of the baits included in the study and the cardiac currents the channels conduct. The antibodies were selected on the basis of MS experiments wherein they captured the channel of interest among the top five most abundant proteins

Gene	Protein	Protein symbol	Class	Current
*Cacna1c*	Ca_v_1.2	Cacna1c	Ca^2+^ channel	*I*_Ca,L_
*Gja1*	Cx43	Gja1	Gap junction channel	*G*_j_
*Hcn4*	HCN4	Hcn4	Funny channel	*I*_f_
*Kcnma1*	BK	Kcnma1	Calcium-activated K^+^ channel	*I*_KCa,2_
*Kcnn3*	K_Ca_2.3/SK3	Kcnn3	Calcium-activated K^+^ channel	*I*_KCa,1_
*Kcnj2*	K_ir_2.1	Kcnj2	Inward rectifier K^+^ channel	*I*_Kir_
*Kcnj3*	K_ir_3.1	Kcnj3	Inward rectifier K^+^ channel	*I*_K,ACh_
*Kcnj5*	K_ir_3.4	Kcnj5	Inward rectifier K^+^ channel	*I*_K,ACh_
*Kcnh2*	K_v_11.1/ERG	Kcnh2	Delayed rectifier K^+^ channel	*I*_K,r_
*Kcna5*	K_v_1.5	Kcna5	Shaker-related K^+^ channel	*I*_Kur_
*Kcnd2*	K_v_4.2	Kcnd2	Subfamily D K^+^ channel	*I*_to1_
*Kcnq1*	K_v_7.1/Kcnq1	Kcnq1	Delayed rectifier K^+^ channel	*I*_K,s_
*Scn5a*	Na_v_1.5	Scn5a	Na^+^ channel	*I*_Na_

**Fig. 2:**
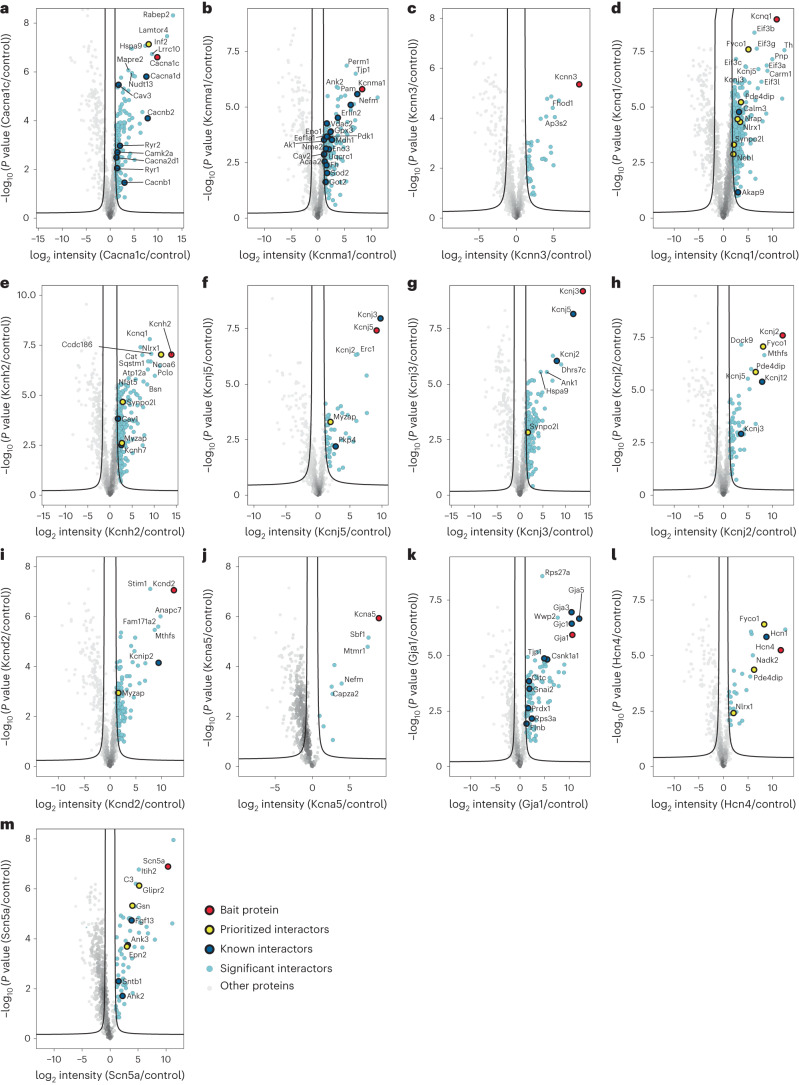
Volcano plot representation for analysis of significant interactors for each channel bait. Volcano plots for each ion channel bait. **a**, Cacna1c. **b**, Kcnma1. **c**, Kcnn3. **d**, Kcnq1. **e**, Kcnh2. **f**, Kcnj5. **g**, Kcnj3. **h**, Kcnj2. **i**, Kcnd2. **j**, Kcna5. **k**, Gja1. **l**, Hcn4. **m**, Scn5a. All dots represent a protein, where the negative logarithm (base 10) of *t*-test-derived *P* value is shown as a function of logarithmic (base 2) ratios of protein intensities in bait pulldowns relative to controls. The control comparator is based on median protein intensities across 64 IP experiments, IgG pulldowns and scaled proteome measurements as well as imputation, and the black line indicates an FDR-based cutoff that considers the fold change difference of protein intensities to demarcate the specific from nonspecific interactors. The claim to significance was based on FDR of a two-sided *t*-test and s0 value (s0 controls the relative importance of *t*-test-based *P* value and difference between means). For details, see Extended Data Fig. 2b and Supplementary Table 2. Proteins shown as light-blue dots represent specific interactors for the bait, red dot is the bait protein itself, dark-blue dot represents interactors with previously reported functional influence on the bait, and yellow dots are protein interactors that we have prioritized for functional investigations based on evaluation of the acquired MS data. Source data

**Extended Data Fig. 2:**
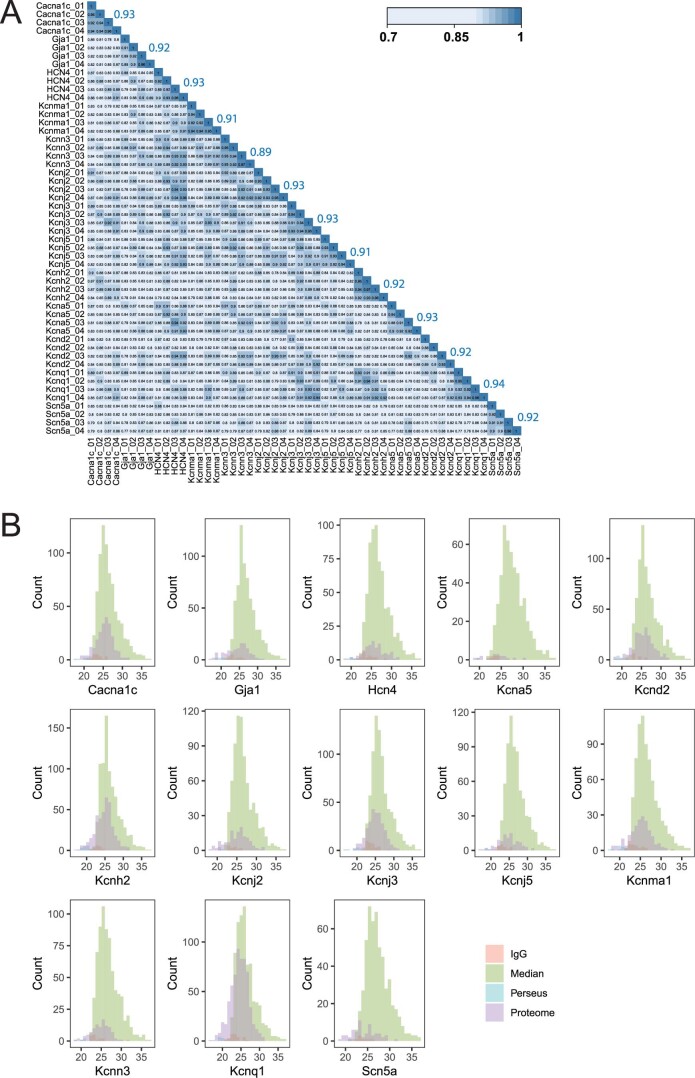
Correlation coefficients and imputation strategy for mass spectrometry data. **a**) Heat map visualizing Pearson correlation coefficients for all measured protein intensities across all experiments. The experiments were carried out in quadruplicates (named as _01, _02, _03, _04) and the figure shows the Pearson correlation values between all the baits and their replicates calculated from the log2 transformed raw intensities of all identified proteins. The darker shade of blue indicates a higher Pearson correlation. The numbers in blue on the right side depict the mean Pearson correlation coefficient for each set of four replicates per bait IP. **b**) Distributions showing the source of protein intensities used in the comparative analysis of each control. As seen, most data come from the median of all measured IP experiments. If no value was present in the median value, a value was used from control IgG pulldown experiments. If no value was available from both the above sources, a scaled proteome measurement was imputed. And in the few cases where no value was obtainable from the proteome either, the value was imputed from a left shifted normal distribution. Source data

The experimental reproducibility and specificity were assessed by unsupervised hierarchical clustering of all measured protein intensities (Fig. 1b). In Fig. 1b, the clustering dendrogram reflects the biological replicate experiments. The four replicates of each IP had a high degree of similarity, which is also reflected in mean Pearson correlation coefficients of 0.92 (Fig. 1c and Extended Data Fig. 2a). The total number of proteins identified per IP ranged from 531 to 1,488 (Supplementary Table 2). In all cases, the channel baits were among the most abundant proteins in their respective pulldowns (Source Data Fig. 2).

To distinguish significant protein interactors from nonspecific ones, we analyzed bait and control pulldowns and visualized the analysis by volcano plots. For this analysis we utilized information from the high number of IPs analyzed in parallel to correct for nonspecific binders (Extended Data Fig. 2b). For imputation of missing values, we utilized information from proteome measurements that we performed on the lysate samples, an approach adapted from a previous study by us^21^. As shown in Fig. 2, we identified 155 protein interactors for Cacna1c, 89 for Gja1, 28 for Hcn4, 9 for Kcna5, 116 for Kcnd2, 228 for Kcnh2, 61 for Kcnj2, 174 for Kcnj3, 45 for Kcnj5, 146 for Kcnma1, 40 for Kcnn3, 364 for Kcnq1 and 59 for Scn5a (the identity of all interactors is provided in table format in Source Data Fig. 2). The number of protein interactors for Kcnq1, Kcnh2 and Cacna1c matches that previously reported for tissue-specific ion channel networks^7^.

**Extended Data Fig. 3:**
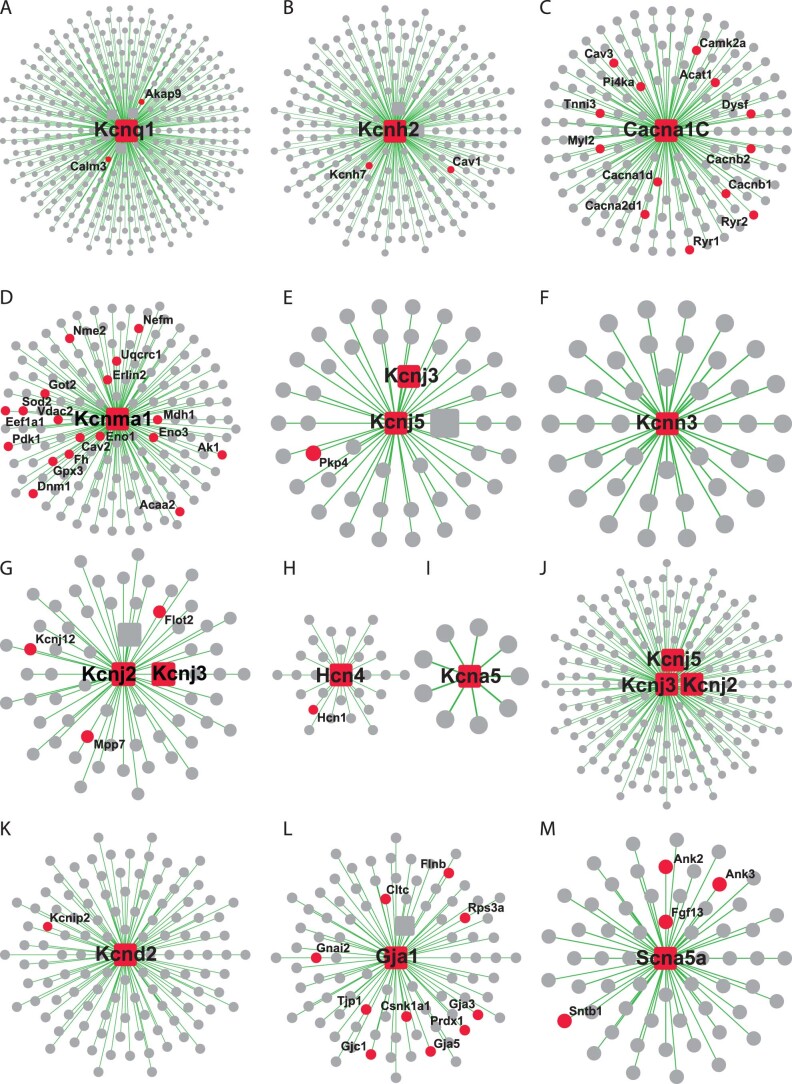
Previously reported interaction partners. Interaction network of **a**) Kcnq1. **b**) Kcnh2. **c**) Cacna1c. **d**) Kcnma1. **e**) Kcnj5. **f**) Kcnn3. **g**) Kcnj2. **h**) Hcn4. **i**) Kcna5. **j**) Kcnj3. **k**) Kcnd2. **l**) Gja1. **m**) Scn5a (baits shown in red squares) with their previously reported interaction partners (shown in red circles). The networks wherein there are more than one red square, the centre protein is the bait. The gray circles/squares represent the novel interaction partners identified in this study.

As an internal validation, our experiment found 59 previously reported protein interactors of the 13 ion channels (Fig. 2 and Extended Data Fig. 3)^8,15,22,23^. For example, we identified the auxiliary subunits Cacnb1 and Cacnb2 of Cacna1c, Fgf13 of Scn5a, Tjp1 of Gja1, Akap9 of Kcnq1, and Flot2 and Mpp7 of Kcnj2. Previous reports^22,24,25^ showed that experiments performed in non-native environments, such as cell lines, are not able to capture cardiomyocyte-specific interactors, which underscores the importance of performing interaction proteomics in the relevant tissue or cellular setting. We attempted reverse IPs for a set of the protein interactors identified, but due to poor antibody specificity (as documented in Supplementary Fig. 1), this strategy was not pursued further.

The dataset presented here greatly expands the current state of knowledge of ion channel interactomes; we performed 64 AP–MS/MS experiments in parallel directly from cardiac tissues and identified 881 protein interactors combined for 13 cardiac ion channels (median of 90 interactors per channel), which represents the most comprehensive mapping of cardiac ion channel interactomes so far.

### Cardiac electrophysiology evaluated in zebrafish KOs

As a first pass to determine the functional effects that different ion channel protein interactors may have on cardiac electrophysiology, we turned to the zebrafish model. This system was favored given that, as opposed to the murine heart (where Kcnq1 does not form a functional channel), the function of the various ion channel proteins can be studied on the same experimental platform. In addition, the zebrafish system has a faster throughput (allowing us to test a higher number of interactors within a reasonable time), and the electrophysiology of the zebrafish heart holds a substantial similarity with that of higher mammals. We systematically knocked out specific genes using a multi-guide RNA clustered regularly interspaced short palindromic repeats (CRISPR)/Cas9 approach in zebrafish embryos^26^ and performed optical voltage mapping on whole hearts isolated from 3–5 days post-fertilization larvae. Sequences of the guide RNAs (gRNAs) used for knockout (KO) in zebrafish are included in Supplementary Tables 3 and 4. After acute KO of the target gene, isolated hearts were incubated with a voltage-sensitive fluorescent dye for optical mapping. Fluorescent signals were first recorded without pacing to capture native conduction for estimation of conduction velocity (CV) and measurement of maximal action potential upstroke velocity (*V*_max_) as well as spatial dispersion of repolarization (standard deviation of repolarization times measured across the chamber from all pixels imaging the chamber). Field pacing was then initiated for the characterization of repolarization including action potential duration (APD) and the spatial dispersion of repolarization at a given pacing rate. The resultant voltage maps were analyzed for atrial and ventricular-specific parameters (Supplementary Tables 5 and 6). Because electrophysiological parameters, especially CV, are highly dependent on the developmental stage of the fish^27^, KOs were compared to unmodified controls from the same clutch. For some genes, KO fish and their wild-type (WT) siblings were also raised until adults and then assessed for differences in their ECG (age at assessment of 8–12 months).

Given the large number of interactors found, we prioritized proteins that were strongly enriched in the precipitate of a given bait, and where the number of peptides identified was greater in the precipitate of the given bait than in that of any of the others. For each channel interactome, the proteins shortlisted by this approach are highlighted in yellow in the volcano plots in Fig. 2. We focused the functional analyses on prioritized interactors for Kcnq1 (Nebl and Nrap), Cacna1c (Inf2) and Scn5a (Glipr2, Epn2 and Gsn) as these channels are the major charge carriers in cardiomyocytes. Note that for Kcnq1 additional protein interactors are prioritized. These are investigated later as they are shared interactors among multiple channels.

**Fig. 3:**
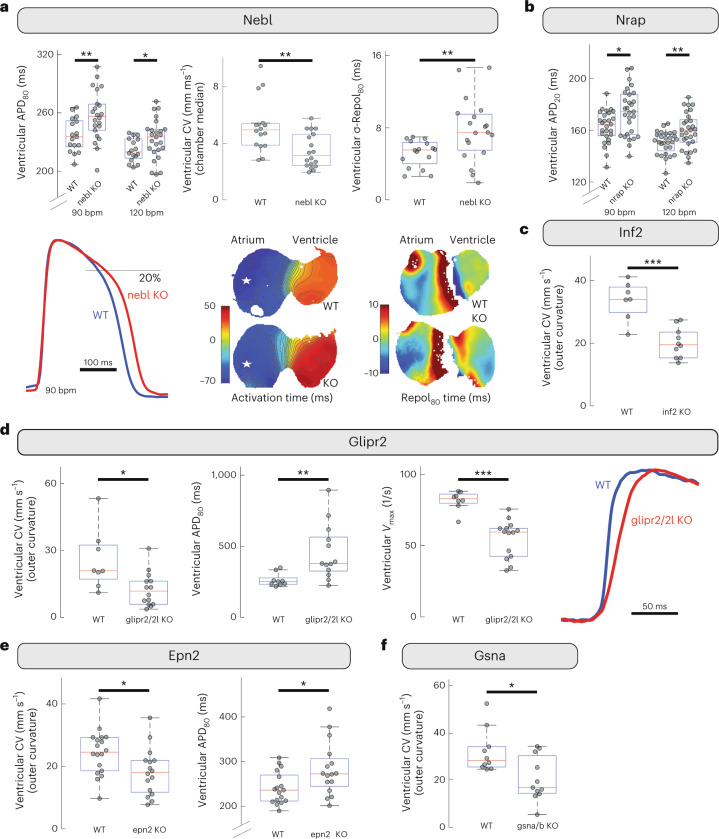
Functional evaluation of channel interactors by gene knock out in zebrafish. The functional consequences after acute KO of six interactors of three major ion channels—Kcnq1 (Nebl and Nrap), Cacna1c (Inf2) and Scn5a (Glipr2, Epn2 and Gsn)—were evaluated in zebrafish. **a**, Compared with control siblings (WT, *n* = 16 fish), KO of the gene encoding Kcnq1-interacting nebl (nebl KO, *n* = 23) led to prolongation of the ventricular action potential starting shortly after the plateau, with the greatest effect size at slower heart rate and later in repolarization (APD_80_, action potential duration measured at 80% recovery; bpm, beats per minute; **P = 8.4 × 10^−3^; *P = 0.037 by two-sided Mann–Whitney *U* test; exemplar amplitude-normalized optical action potentials shown below), reduced ventricular CV (sinus rhythm; **P = 4.2 × 10^−3^ by idem; exemplar relative activation time maps are zero-referenced to activation of the AV-Ring; white stars indicate area of global earliest activation, isochrones denote 5 ms intervals), and increased spatial dispersion of repolarization (σ-Repol_80_, standard deviation of repolarization time at 80% recovery across the chamber; sinus rhythm; **P = 8.74 × 10^−3^, idem; in exemplar relative repolarization_80_ time maps, each chamber is zero-referenced to median repolarization time). **b**, KO of Kcnq1-interacting nrap (*n* = 29) led to prolongation of ventricular action potential with greatest effect size in early repolarization (APD_20_) and at faster heart rates (*P = 0.016; **P = 5.22 × 10^−3^, idem, *n*_WT_ = 27). **c**, Knockdown of Cacna1c-interacting inf2 (*n* = 10) caused a significant decrease in ventricular CV (***P = 7.2 × 10^−4^, idem, *n*_WT_ = 7). **d**, KO of Scn5a-interacting glipr2/glipr2l (*n* = 14) decreased ventricular CV (*P = 0.013) and rate of the action potential upstroke (*V*_max_; ***P = 2.61 × 10^−4^, exemplar amplitude normalized action potential upstrokes shown) as well as increasing ventricular APD (**P = 2.7 × 10^−3^, all by idem, *n*_WT_ = 9). **e**, KO of Scn5a-interacting epn2 (*n* = 16) resulted in decreased ventricular CV (*P = 0.020) and increased ventricular APD_80_ (*P = 0.035, idem, *n*_WT_ = 18). **f**, Decrease in ventricular CV was also observed after KO of Scn5a-interacting gsna/b (*n* = 11, *P = 0.027, idem, *n*_WT_ = 10). Each point in the box plots corresponds to an individual zebrafish embryo. Box plots indicate 25th/50th/75th percentiles, while whiskers extend to the most extreme data within 1.5× of interquartile range beyond box limits. Source data

**Extended Data Fig. 4:**
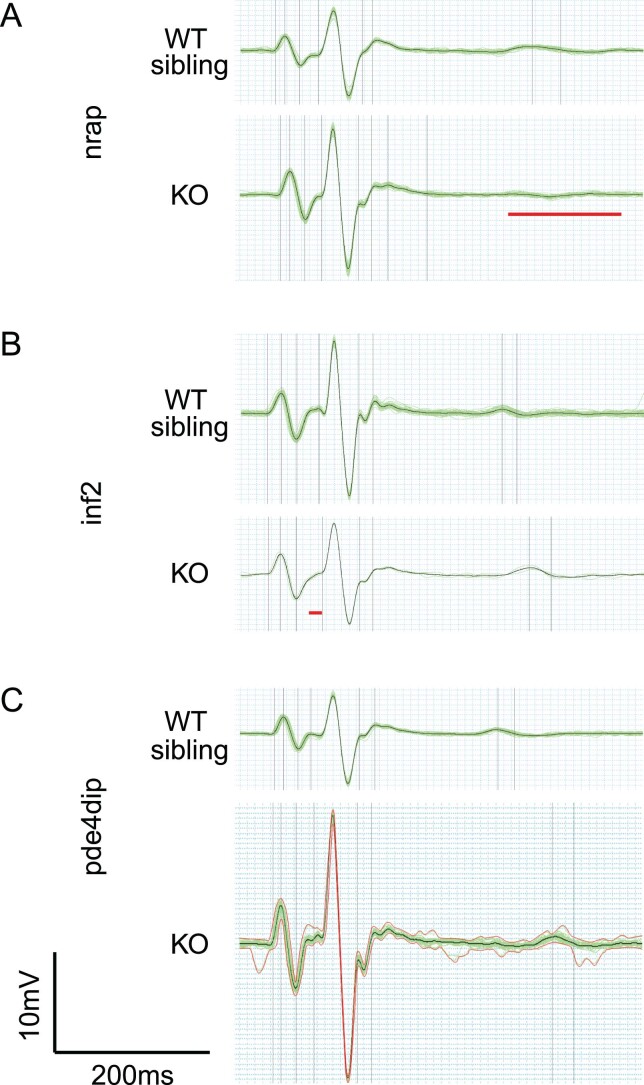
Exemplar ECGs from adult zebrafish gene knockouts (KO) and their wildtype (WT) siblings. Signal-average trace (black) overlaid R-aligned traces from individual beats (green). **a**) Nrap knockout ECGs displayed repolarization abnormalities such that T-waves were not discernible from signal noise (red underscored period). **b**) ECGs from inf2 knockout fish had continued rising activity throughout the PR segment without achieving an isoelectric level as typically seen in wildtype or other knockouts (red underscored period). **c**) ECGs from pde4dip knockout fish displayed greater variance in signal waveform between individual beats showing substantial deviations from mean well in excess of typical noise (extrema envelope in red).

The Kcnq1 protein interactors Nebl and Nrap belong to the nebulin family and are involved in actin binding and anchoring^28,29^. The Kcnq1 channel acts as a heart-rate dependent carrier of repolarizing current. Accordingly, acute KO in zebrafish of the gene encoding the Kcnq1 interactor, nebl, led to a significant prolongation of the ventricular action potential starting in early phase-3 repolarization (shortly after the action potential plateau phase), and becoming more obvious in later stages of repolarization, with a greater prolongation effect size at slower heart rates (Fig. 3a and Supplementary Table 6). We also observed a decrease in CV and increased spatial dispersion of repolarization during sinus rhythm in these hearts (Fig. 3a and Supplementary Tables 5 and 6). Similarly, KO of the gene encoding the Kcnq1 interactor nrap also prolonged ventricular phase-3 repolarization, with greatest effect size early in repolarization and at faster heart rates (Fig. 3b and Supplementary Table 6). Adult zebrafish with KO of the gene encoding nrap displayed repolarization abnormalities in the ECG T-waves (Extended Data Fig. 4a and Supplementary Table 7). Phenotypes of KOs of the genes encoding both nebl and nrap are consistent with effects on the rate-sensitive delayed rectifier current arising from Kcnq1.

Regarding inward currents, KO of the gene encoding inf2 (part of the Cacna1c protein network) and of the genes encoding each of the prioritized Scn5a interactors (glipr2/glipr2l, epn2 and gsna/gsnb) caused a significant decrease in ventricular CV (Fig. 3c–f and Supplementary Table 5), consistent with a role for these interactors in maintaining cell excitability. KO of the genes encoding glipr2/glipr2l and epn2 also led to a significant increase in ventricular APD. In the adult zebrafish, KO of the gene encoding inf2 displayed continued activity throughout the PR segment and a general increase in the PR interval consistent with slowed conduction through the atrioventricular (AV) canal (Extended Data Fig. 4b and Supplementary Table 8).

Taken together, our results show that the interactors identified via AP–MS/MS can regulate cardiac electrophysiology. The functional effect is probably mediated via their interaction with the respective ion channels: Kcnq1 (Nebl and Nrap), Cacna1c (Inf2) and Scn5a (Glipr2, Epn2 and Gsn).

### Epsin-2 and gelsolin regulate sodium current in myocytes

**Extended Data Fig. 5:**
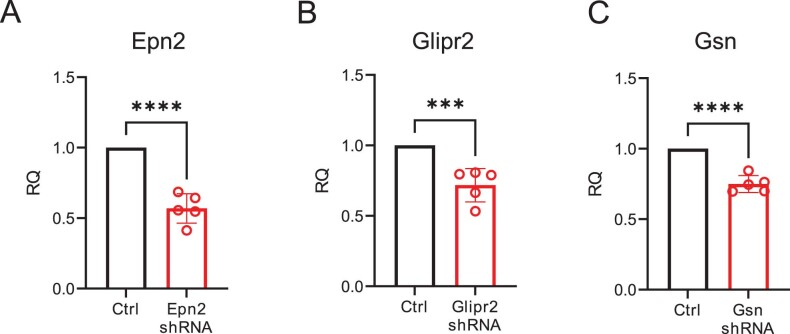
Real Time PCR of Epn2, Glipr2 and Gsn from murine cardiomyocytes. Transcript abundance for Epn2 (**a**), Glipr2 (**b**) and Gsn (**c**) in hearts of AAV9-shRNA injected mice were compared to that of GAPDH (control). Relative Quantification (RQ) was calculated as 2^−^CT and normalized to control mice injected with empty AAV-EGFP vector. Statistical significance was calculated by two-sided Student’s t test, no adjustments were made for multiple comparisons. The RNA samples were obtained from five AAV-shRNA injected mice for each gene. (n = 5 mice for each condition; **** P < 0.0001; *** P = 0.0007; Data are presented as mean values +/− SD’). Source data

After functional validation in the homogeneous platform of the zebrafish system, we turned our attention back to the murine heart. Given that the sodium current is functionally preserved across species, we focused our attention on the interactors of Scn5a and their possible role in determining the magnitude and voltage-gating characteristics of the current. Expression of each one of the three lead Scn5a interactors (Glipr2, Epn2 and Gsn) was knocked down, one at a time, using specific short hairpin RNA (shRNA) constructs, delivered via adeno-associated viral vectors (AAVs) that were injected in the tail vein of adult mice. All constructs were bicistronic and included the coding of eGFP; fluorescence from the GFP reporter was used to select cells for patch clamp study. We included two control groups: cells collected from uninjected mice, and cells from mice injected with an AAV vector containing only eGFP. Patch clamp experiments were performed 17–21 days after AAV injection. Specifics on silencing of the genes encoding Glipr2, Epn2 or Gsn are noted in Extended Data Fig. 5.

**Fig. 4:**
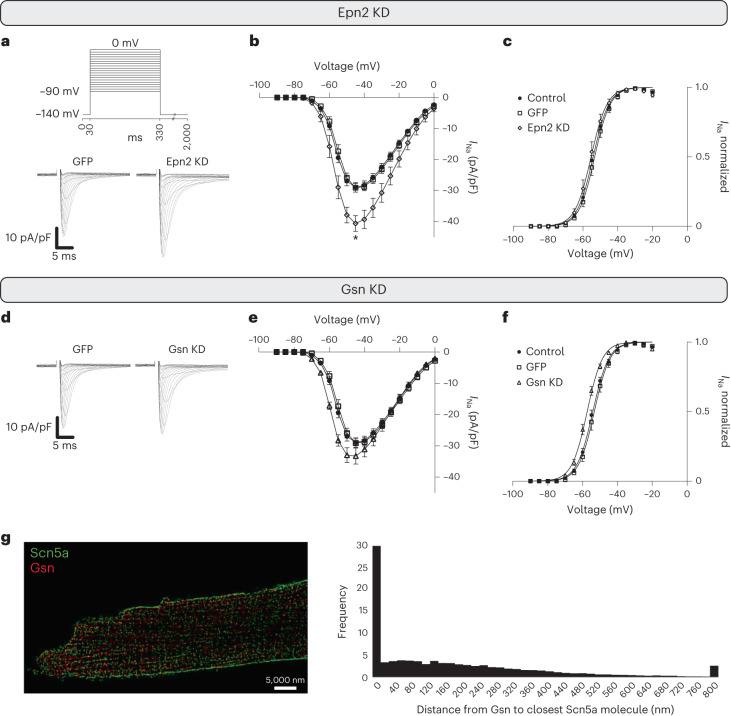
shRNA silencing of sodium channel interactors, Epn2 and Gsn, increase sodium current density in mouse ventricular cardiomyocytes. **a**, Voltage clamp protocol (top) and representative sodium current (*I*_Na_) traces measured from adult cardiomyocytes expressing only GFP (GFP; bottom left) or GFP as well as shRNA for Epn2 (Epn2 knockdown (KD); bottom right). **b**, Current (*I*) to voltage (*V*_m_) relationship of *I*_Na_ obtained from cardiomyocytes that were not injected (‘control’; solid circles), expressing only GFP (GFP; open squares) or from expressing GFP and shRNA for Epn2 (Epn2 KD; open diamonds). The data show increased peak sodium current density in Epn2 KD (*P = 0.014, linear mixed-effects analysis followed by Bonferroni post hoc analysis for multiple comparison testing). **c**, Sodium current activation measured for Epn2 KD, GFP or uninjected controls. **d**, Representative *I*_Na_ traces in GFP-expressing cardiomyocytes (bottom left) and in Gsn2 KD cardiomyocytes (bottom right). **e**, *I* to *V*_m_ relationship of *I*_Na_ for Gsn KD cardiomyocytes compared to that of controls (maximum sodium current trending to be increased for Gsn KD, *P* = 0.181, linear mixed-effects analysis followed by post hoc Bonferroni correction). **f**, *I*_Na_ activation curves. The activation curve is negatively shifted in Gsn KD cardiomyocytes compared to that of myocytes expressing only GFP (*V*_1/2,Gsn KD_ = −57.4 ± 0.63 mV; *V*_1/2,GFP_ = −53.4 ± 0.95 mV, *P = 0.037 linear mixed-effects analysis, followed by Bonferroni post hoc analysis for multiple comparison tests, control: *n* = 10 cells obtained from 3 mice; GFP: *n* = 9 cells obtained from 3 mice; Epn2 KD: *n* = 9 cells obtained from 4 mice; Gsn KD: *n* = 8 cells obtained from 4 mice; data are presented as mean ± standard error of the mean). **g**, Two-color STORM images for Scn5a (green) and Gsn (red) show 30% of Gsn clusters localizing within 20 nm of Scn5a clusters, 15 cells obtained from 3 mice in independent experiments. ‘Control’ are cardiomyocytes isolated from WT animals. ‘GFP’ are cardiomyocytes isolated from animals injected with an empty AAV vector. ‘Epn2 KD’ are cardiomyocytes isolated from mice with *Epn2* silencing and ‘Gsn KD’ from mice with *Gsn* silencing. Multiple animals per group were necessary due to the limited number of datapoints that can be obtained from a single animal. Source data

**Extended Data Fig. 6:**
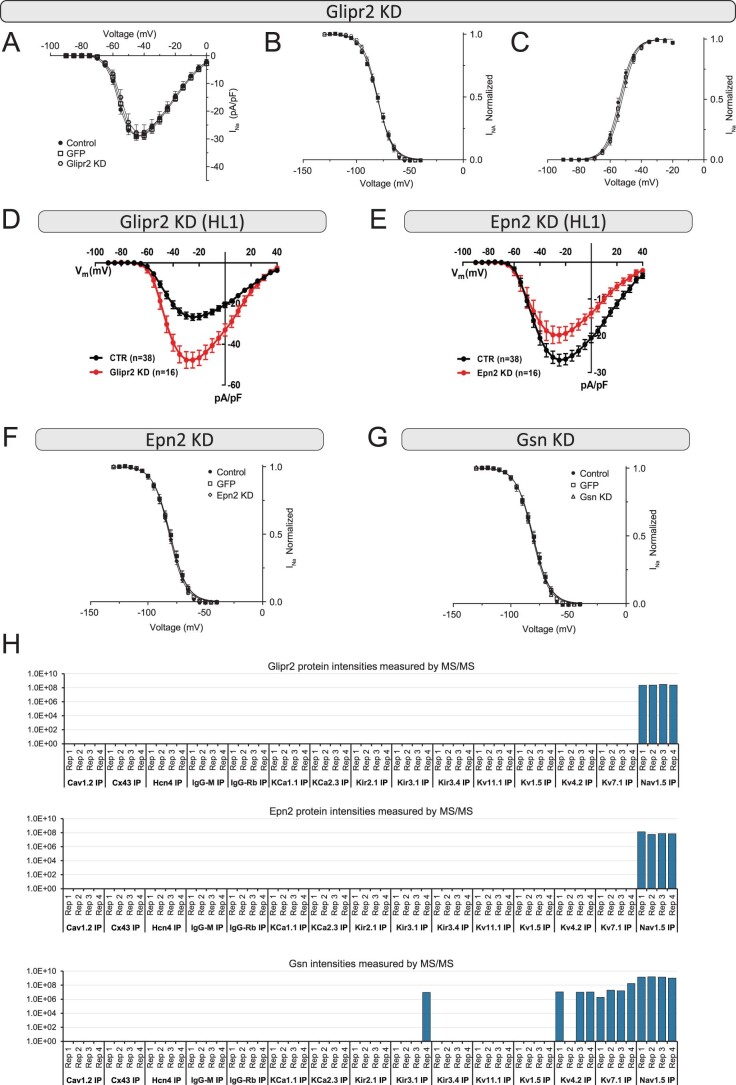
Effects ion electrophysiological parameters upon knock down of novel sodium channel interactors. Cardiomyocytes were harvested from mice that were either non-injected (control; closed circles), injected with AAV9 containing the GFP coding region (open squares) or with AAV9 containing the coding region for GFP, as well as shRNA to knockdown (KD) the protein of interest. Multiple animals per group were necessary due to the limited number of data points that can be obtained from a single animal. (**a**) Glipr2 was evaluated. Glipr2 KD in cardiomyocytes had no effect on sodium current density (control, n = 10 cells obtained from 3 mice; GFP, n = 9 cells obtained from 3 mice; Glipr2 KD, n = 7 cells obtained from 4 mice). (**b**) Steady-state voltage dependence of inactivation (control, n = 10 cells obtained from 3 mice; GFP, n = 8 cells obtained from 3 mice; Glipr2 KD, n = 9 cells obtained from 4 mice) and activation (**c**) were also unaffected. (**d**) Peak sodium current density recorded from HL1 cells treated with siRNA targeting Glipr2 was larger than that of control. (**e**) Peak sodium current density recorded from HL1 cells treated with siRNA targeting Epn2 was less than that of control cells. Control, n = 38 cells; Glipr2 KD, n = 16 cells; Epn2 KD n = 16. (**f–g**) The steady-state voltage dependence of inactivation of INa measured were unaffected by reduced expression of Epn2 (F) or Gsn (G), measured in adult murine cardiomyocytes harvested from mice injected with AAV9 (control n = 10 cells obtained from 3 mice; GFP n = 8 cells obtained from 3 mice; Epn2 KD n = 8 cells obtained from 4 mice; Gsn KD n = 10 cells obtained from 4 mice). Data are presented as mean values +/− SEM. (**h**) Protein abundances of Glipr2 (top), Epn2 (middle) and Gsn (bottom) measured across replicate immunoprecipitations as outlined from murine cardiac tissue. Glipr2 and Epn2 were exclusively identified in the four Scn5a (Nav1.5) replicates. Gsn was consistently measured at high abundance in the four Nav1.5 immunoprecipitations but was also present in the Kcnq1 (Kv7.1) immunoprecipitations as well as in some of the Kcnd2 (Kv4.2) immunoprecipitations. Gsn was more than a hundred-fold more abundant in the Nav1.5 IPs. Source data

Silencing *Glipr2* did not impact the sodium current recorded from adult mice cardiomyocytes (Extended Data Fig. 6a–c), although we observed that knockdown of *Glipr2* in HL1 cells led to increased sodium current density (Extended Data Fig. 6d). AAV-delivered shRNA silencing of *Epn2* on the other hand led to a significant increase of the sodium current in adult murine cardiomyocytes (Fig. 4a,b). The latter was consistent with the fact that reduced expression of epn2 in zebrafish alters sodium current density, though in the murine heart, the direction of change was reversed. The relation between Epn2 expression and *I*_Na_ function was also demonstrable in HL1 cells, though in that system current density was also reduced after *Epn2* knockdown (Extended Data Fig. 6f). The increase in sodium current amplitude in the adult cardiomyocytes in absence of Epn2 hence appears to be particular for fully differentiated cardiomyocytes. Of note, no *Epn2* knockdown-dependent changes in the steady-state voltage dependence of *I*_Na_ were observed (Fig. 4c and Extended Data Fig. 6e). Similar to *Epn2* silencing, silencing of *Gsn* trended to increased maximum sodium current in adult cardiac myocytes (Fig. 4d,e). Silencing of *Gsn* had no significant impact on sodium channel inactivation (Extended Data Fig. 6g) but did lead to a left-shift of the steady-state voltage dependence of activation (Fig. 4f), contributing to increased sodium current in cardiomyocytes absent of Gsn. Epn2 and Glipr2 were only identified by IP of Scn5a, whereas Gsn was identified in two other channel pulldowns, though its abundance in those precipitates was a hundred times less than in the Scn5a pulldown (Extended Data Fig. 6h). To further evaluate the interaction network containing Scn5a and Gsn, we performed two-color super-resolution microscopy (stochastic optical reconstruction microscopy, STORM) in cardiomyocytes isolated from WT adult mice. Using an antibody specific for Gsn, we found that 30% of Gsn clusters localized within 20 nm of Scn5a clusters in adult cardiomyocytes (Fig. 4g and Supplementary Fig. 2). The electrophysiology results obtained from adult murine cardiomyocytes support a functional role for two of the three interaction partners evaluated; in particular the results show that absence of one of the two interaction partners, Epn2 and Gsn, leads to increased sodium current density in adult cardiomyocytes.

### Interactors shared between multiple ion channels

**Fig. 5:**
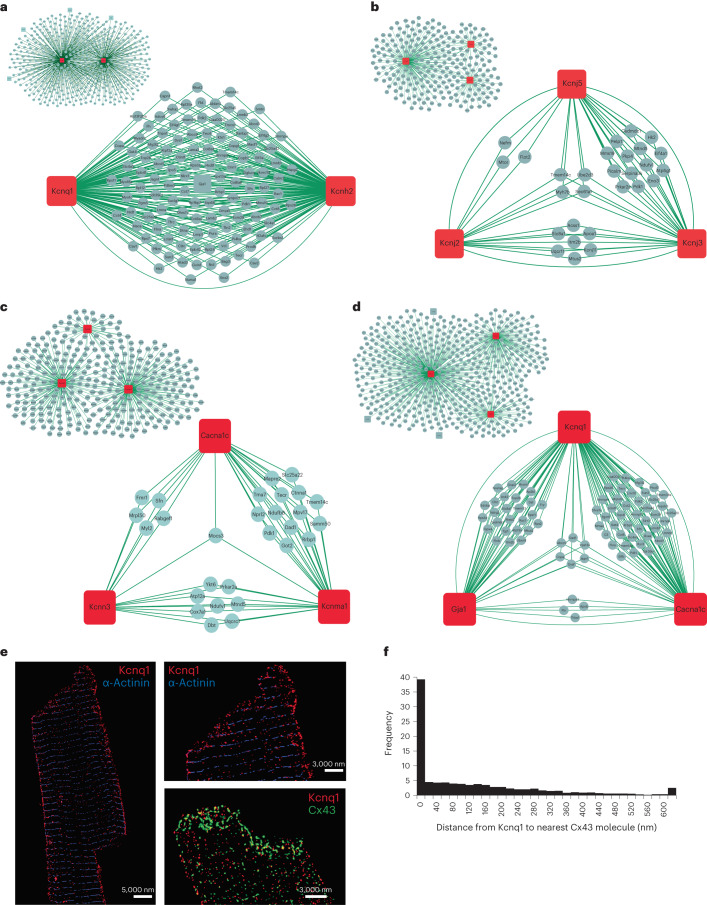
Inter-channel networks. Networks of shared proteins between channels found to interact. **a**–**d**, Kcnq1 and Kcnh2 (**a**), between Kcnj2, Kcnj3 and Kcnj5 (**b**), between Cacna1c, Kcnn3 and Kcnma1 (**c**), and between Kcnq1, Gja1 and Cacna1c (**d**). The inset panels show all shared interactors for these channels. The bait proteins are shown in red squares and the interactors in light-blue circles. Measured interactions are indicated by lines. Note the interactions between channel proteins. **e**, STORM images of murine cardiomyocytes for Kcnq1 (red) and Gja1 (green). α-Actinin shown in blue as a control. **f**, Quantification of images as those shown in **e** shows that 40% of Kcnq1 clusters localize within 20 nm from Gja1 clusters. Twenty cells were examined over three mice in independent experiments. Source data

**Extended Data Fig. 7:**
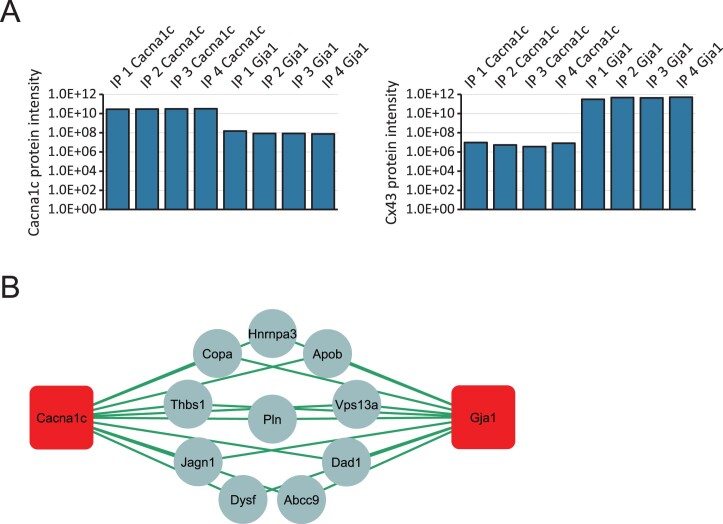
Shared interactome of Cacna1c and Gja1. **a**) Protein abundances of Cacna1c (Cav1.2) and Gja1 (Cx43) measured by mass spectrometry evaluation of quadruplicate immunoprecipitations of Cacna1c (left) and of Gja1 (right) from murine cardiac tissue. Cav1.2 was precipitated by the Cav1.2 IPs as well as by the Cx43 IPs. Similarly, Cx43 was immunoprecipitated by the Cx43 IPs as well as by the Cav1.2 IPs. **b**) Protein interaction network outlining the 10 shared interactors we identified for the networks of Cacna1c and Gja1.

In the section above, we investigated the functional impact of proteins we identified by AP–MS/MS to be part of the protein interaction network of the cardiac sodium channel. However, other interactors were found associated with more than one ion channel protein. In fact, for each channel there were two populations of interactors: one consisting of proteins pulled down only by that single bait, and another one consisting of proteins that were pulled down by more than one bait. In several cases, ion channel proteins appeared, themselves, as precipitates not only of the antibody for which they were a target, but also as interactors of a different channel, indicating that ion channel proteins share a notable fraction of their molecular partners. Figure 5 illustrates the proteins found in four such shared networks (the information is available in table format in Source Data Fig. 5). The first network is formed by two potassium-selective ion channels known to physically interact in cardiomyocytes^30^ (Kcnq1/Kcnh2; Fig. 5a), a second one is formed by inward rectifiers (Kcnj2/Kcnj3/Kcnj5; Fig. 5b), a third one is formed by channels whose activity depend on intracellular calcium concentrations (Kcnma1/Kcnn3/Cacna1c; Fig. 5c) and a fourth network results from the convergence of Gja1/Cacna1c/Kcnq1 interactors (Fig. 5d). Cacna1c and Gja1 are known to co-localize in cardiomyocytes^31^. Here we present evidence that Cacna1c and Gja1 co-precipitate in IPs and have a shared protein interaction network (Extended Data Fig. 7). Their potential co-localization with Kcnq1 is interesting as Kcnq1 appears sensitive to intracellular calcium homeostasis^32^. We tested the possibility of co-localization of Gja1 and Kcnq1 in adult myocytes by super-resolution microscopy. α-Actinin was used as a marker of the *z*-discs, and Gja1 marked the cell end (Supplementary Fig. 3). Panel 5E shows the position of Kcnq1 and α-actinin as well as that of Kcnq1 and Gja1. There is notable co-localization, at the cell end, of Kcnq1 and Gja1 (Fig. 5f). These data suggest that Kcnq1, and its interactome, may be in physical proximity to Gja1 at the intercalated disc. The shared channel interactors presented in Fig. 5 argue that several of the cardiac ion channel networks intersect with one another, and that they are probably clustered physically at shared locations in the cardiomyocyte.

### Shared interaction partners also affect electrophysiology

**Fig. 6:**
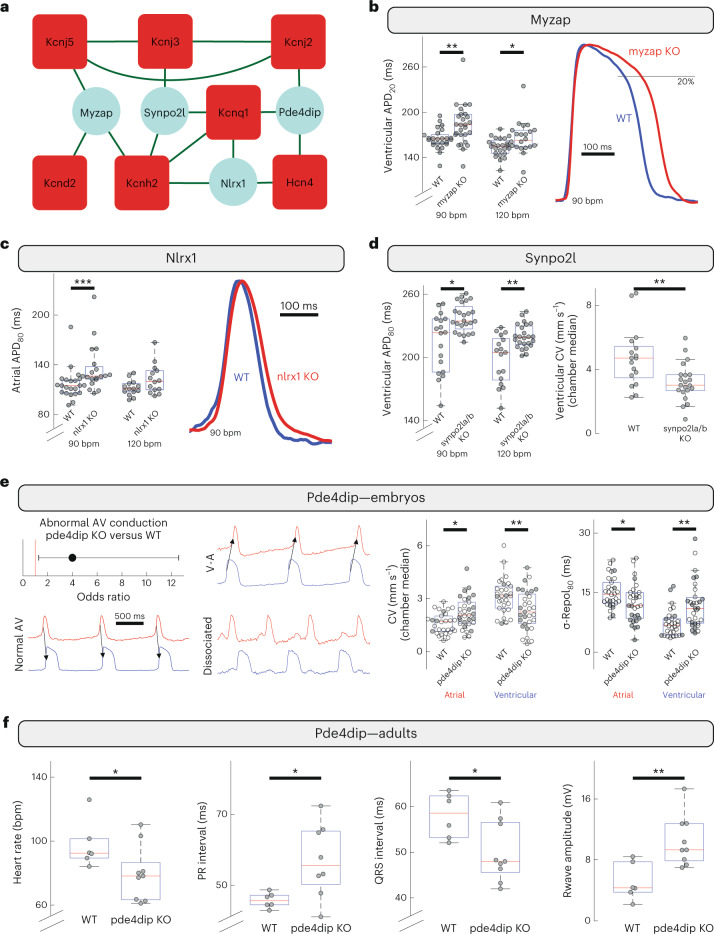
Functional evaluation of interactors shared across multiple channel networks. Four proteins that each interact with three different ion channels were functionally investigated. **a**, Interactions identified for Myzap, Nlrx1, Pde4dip and Synpo2l. Bait proteins are shown in red, interactors in light blue and interactions in green. **b**, Acute KO in zebrafish of the gene encoding myzap (*n* = 25 fish) resulted in prolonged ventricular APD (APD_20_; **P = 2.85 × 10^−3^; *P = 0.018 by two-sided Mann–Whitney *U* test; exemplar amplitude-normalized optical action potentials shown) compared to control siblings (WT, *n* = 25). **c**, KO of nlrx1 (*n* = 21) mainly affected atrial APD, with a greater effect size in late repolarization (APD_80_) and at slower heart rates (*n*_WT_ = 21, ***P = 7.64 × 10^−4^ by idem; exemplar amplitude normalized optical action potentials shown). **d**, KO of synpo2la/b (*n* = 23) led to prolonged ventricular APD at multiple paced heart rates with greatest effect size late in repolarization (*n*_WT_ = 17, *P = 0.014; **P = 5.04 × 10^−3^, idem) and reduced ventricular CV (sinus rhythm; **P = 0.015, idem). **e**, KO of pde4dip (*n* = 30) resulted in hearts more prone to abnormal AV conduction (*n*_WT_ = 30, odds ratio and 95% CI shown; *P* = 0.029 by Fisher’s exact test; exemplars show normal atrioventricular conduction versus retrograde conduction or AV dissociation), decreased ventricular CV and increased spatial dispersion of ventricular repolarization (standard deviation of repolarization time across the chamber), but with inverted effects in the atrial chamber (intrinsic rhythm; CV: *P = 6.24 × 10^−3^; **P = 5.86 × 10^−3^; σ-Repol_80_: *P = 0.013; **P = 1.66 × 10^−4^, idem). This reduces the differential between the chambers, which was observed in pde4dip-deficient fish with both abnormal AV conduction (filled markers) and normal (open markers). This suggests episodic abnormal AV conduction resulting in electrical remodeling with persisting effects during periods of normal AV conduction. **f**, In adult zebrafish, pde4dip deficiency (*n* = 9) resulted in slower heart rate (*P = 0.049), longer PR and shorter QRS intervals (*P = 0.029 and *P* = 0.036, respectively), and greater R wave magnitude (**P = 0.0095, all by idem, *n*_WT_ = 6). Each point in the box plots corresponds to an individual zebrafish. Box plots indicate 25th/50th/75th percentiles, while whiskers extend to the most extreme data within 1.5× of interquartile range beyond box limits. Source data

The protein networks depicted in Fig. 5 illustrate that several molecular components of ion channel networks interact with more than one ion channel. However, as protein complexes are dynamic and can be quite extensive, it is unclear if these multiple ion channel interactors contribute meaningfully to proper ion channel homeostasis or are simply distal members of the greater complex with minimal impact on electrophysiology. To evaluate if these proteins that interact with multiple ion channels can be functionally relevant to electrophysiological properties, we pursued functional investigation of a subset of them. Specifically, we focused on four interactors that associate with three different channel proteins (yellow dots in Fig. 2, Fig. 6a): Myzap is in the protein network of channels Kcnj5/Kcnd2/Kcnh2; Nlrx1 is in the protein network of channels Kcnq1/Kcnh2/Hcn4; Pde4dip is in the network of channels Kcnj2/Kcnq1/Hcn4; and Synpo2l is in the network of channels Kcnj3/Kcnq1/Kcnh2. As in the case of the single interactors, the zebrafish model was used as experimental platform, given, among other reasons, the lack of electrically relevant Kcnq1 expression in the murine heart.

**Extended Data Fig. 8:**
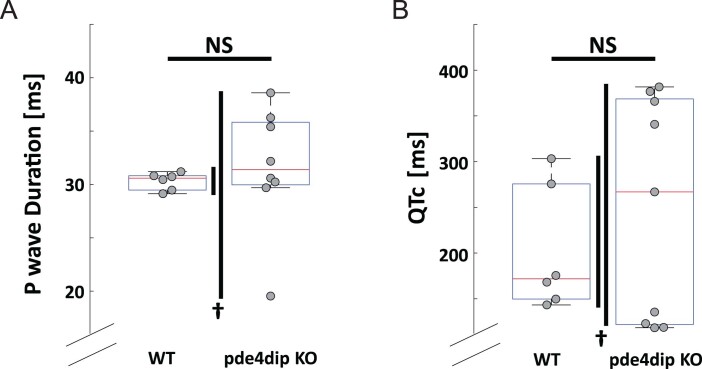
Greater ECG parameter variability in adult pde4dip knockout (KO) zebrafish compared to wildtype siblings (WT). Some ECG parameters had similar median values between WT (n = 6 fish) and pde4dip knockout (n = 9) groups (NS p > 0.05); however, the pde4dip group had much greater variance in P wave duration (**†** p = 0.013; panel **a**) and corrected QT interval (**†** p = 0.014, both by 2-sided Conover squared-ranks test for homogeneity of variance; panel **b**). Each point in the boxplots corresponds to an individual zebrafish. Boxplots indicate 25^th^/50^th^/75^th^ percentiles, while whiskers extend to the most extreme data within 1.5x of interquartile range beyond box limits. Source data

Using multi-gRNA CRISPR/Cas9 we acutely knocked out the orthologous gene(s) encoding each of the four proteins, Myzap, Nlrx1, Pde4dip and Synpo2l, in zebrafish embryos and evaluated these genetic manipulations by cardiac optical voltage mapping. The results are shown in Fig. 6b–e and Supplementary Table 6. Figure 6b shows that acute KO of the gene encoding myzap led to prolonged ventricular APD that was well established by early phase-3 repolarization, consistent with an effect on an early repolarization current (such as *I*_to_) which arises in part from Kcnd2. KO of the gene encoding nlrx1 prolonged atrial APD, with greater effect size in late repolarization and a strong rate dependency (Fig. 6c). Acute KO of the gene encoding synpo2l caused ventricular APD prolongation with greatest effect late in repolarization, consistent with reduced outward current during mid-to-late phase-3 repolarization consistent with the currents arising from Kcnq1 and Kcnh2 (Fig. 6d). Like loss of nebl, loss of synpo2l resulted in reduced ventricular CV and increased spatial dispersion of ventricular repolarization (Fig. 6d and Supplementary Table 6). In embryonic zebrafish, acute KO of the gene encoding pde4dip resulted in an increased propensity to abnormal AV conduction pattern including AV dissociation and ventricular ectopic pacing foci with retrograde AV conduction. Hearts absent of pde4dip also had slower ventricular CV and increased spatial dispersion of ventricular repolarization, but KO caused these parameters to change in the opposite direction in the atria (Fig. 6e). Adult zebrafish absent in pde4dip had normal antegrade, 1:1 associated AV conduction, but the PR interval was prolonged indicating impaired AV conduction. These fish exhibited additional signs of electrical remodeling such as slower heart rates and altered QRS waves, the latter suggesting changes in ventricular conduction (Fig. 6f and Supplementary Table 8). Additionally, these fish had greater beat-to-beat variability in their ECG waveform and parameters (Extended Data Figs. 4c and 8). Although the specific functional impact of these interactors with each channel separately requires in-depth investigation, these results demonstrate that the associations detected biochemically are in fact necessary for the electrical homeostasis of the heart.

### Interaction partners and ECG phenotypes

**Fig. 7:**
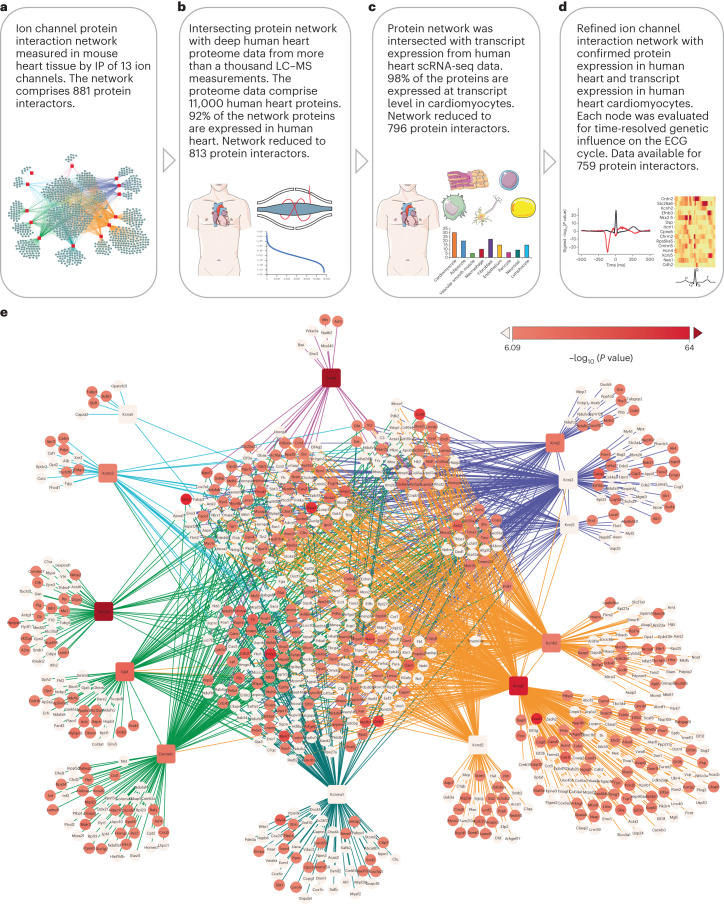
Network nodes associated with genetic influence on human heart ECG. **a**, We constructed a combined protein–protein interaction network of all 13 channel interactomes, which in total comprise 881 protein interactors. Channel bait proteins are shown in red squares, interactors in gray-blue circles. Edges are colored to indicate clusters of ion channels that contribute to similar electrophysiological components. **b**, The network from **a** was filtered for proteins that were measured in human heart samples by analyzing more than a thousand MS-based proteomics measurement files from human heart samples. Ninety-two percent of the proteins in the network were identified in the human heart samples (details in Supplementary Fig. 4a and Supplementary Table 9). **c**, We utilized human heart single-nucleus RNA sequencing data to determine which of the interactors were expressed in human cardiomyocytes. We found evidence of expression for 98% of the interactors (details in Supplementary Fig. 4b and Supplementary Table 9). **d**, The remaining 796 human heart, cardiomyocyte-expressed, interactors were evaluated using ECG plotter tool. For each protein, this generates a time series of associations across the ECG cycle. For each protein, we report the most significant association. **e**, Refined network of the 13 channel bait proteins and their human heart-cardiomyocyte-expressed interactors. Bait proteins are depicted in squares, interactors in circles. The color of the nodes indicates the significance of the influence on the ECG as determined by ECG plotter. A darker red color indicates a more significant association. The 340 proteins with a significant influence (*P* < 8.23 × 10^−7^ resulting network supports the notion that the combined ion channel network is enriched for proteins that influence the cardiac ECG. GWAS *P* values were extracted from Verweij et al.^33^ and adjusted for multiple comparisons (details in Methods and Source Data Fig. 7). Parts of the figure were drawn by using pictures from Servier Medical Art. Servier Medical Art by Servier is licensed under a Creative Commons Attribution 3.0 Unported License (https://creativecommons.org/licenses/by/3.0/). Source data

**Extended Data Fig. 9:**
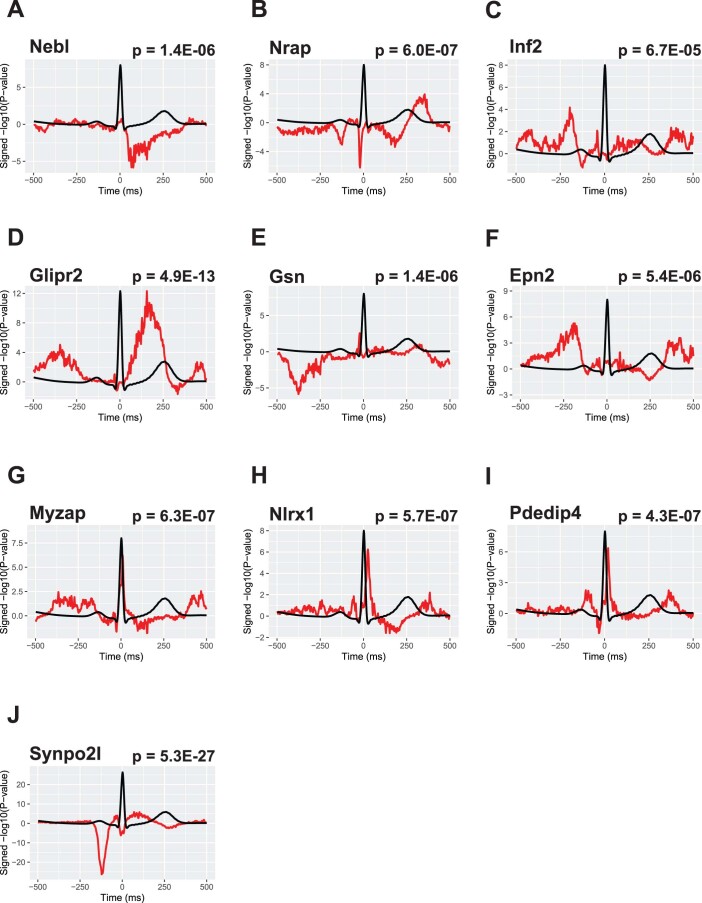
Genetic ECG signatures of the key interactors of cardiac ion channels identified in this study and studies using zebrafish acute knockouts. Genetic ECG signatures for **a**) Nebl, **b**) Nrap, **c**) Inf2, **d**) Glipr2, **e**) Gsn, **f**) Epn2, **g**) Myzap, **h**) Nlrx1, **i**) Pde4dip and **j**) Synpo2l with their respective maximum p-values shown on the upper right corner. The black line shows the average ECG curve of the full cohort used by Verweij et al.^33^, centered at the R wave ± 500 ms. The red line depicts the -log10(p value) of the association (signed to show the direction of association) for each of the 500 spatiotemporal data points of the ECG curve with the ECG morphology phenotype, that is, showing the strength of association of the genetic loci of a gene with each 2 ms window of the ECG. GWAS P-values were extracted from Verweij et al.^33^ and adjusted for multiple comparisons (see methods for details).

All ion channels investigated herein contribute to the cardiac electrical cycle. We have confirmed in zebrafish KOs that a subset of the interactors we have identified have a measurable impact on the proper homeostasis of these sarcolemmal ion channels, including some interactors where the functional contribution is not defined, for example, Nlrx1, which canonically is involved in mitochondrial antiviral response. We have also shown that two of the interactors influence sodium current in adult cardiomyocytes. We next set out to evaluate which of the newly identified ion channel interactors are most likely to impact human cardiac electrophysiology. To do so, we applied a multi-omics data integration strategy. First, we utilized a human population genetics resource developed by Verweij et al.^33^, where the influence of genetics on the high-dimensional representation of the ECG was mapped. Instead of focusing on the individual segments of the ECG, Verweij and colleagues performed a comprehensive phenotyping of more than 77,000 ECGs in the UK Biobank across the complete cycle of cardiac conduction, resulting in 500 spatial–temporal datapoints across 10 million genetic variants, describing the genetic signatures of the ECG. As an example of the information contained in the ECG GWAS dataset, we present the genetic ECG signatures of the ten proteins we have functionally investigated in Extended Data Fig. 9. Nine of the ten proteins are encoded in genetic loci significantly associated with an ECG phenotype in humans. We utilized the genetic resource to address, in a global manner, which of the identified interactors are likely to contribute to the human heart ECG (Fig. 7).

We first evaluated which of the network proteins (nodes) are present in the human heart and which are expressed in the relevant cell type, the cardiomyocyte. For this, we integrated the interactome data from all 13 channels into a global protein interaction network (Fig. 7a). Next, we utilized information from more than a thousand deep proteomics measurements of human heart samples, many of which were collected by us, to evaluate whether the interactors identified in murine hearts are also expressed at the protein level in human hearts (Fig. 7b). This analysis showed that more than 92% of the proteins in the ion channel network were identified in our human cardiac proteome atlas (Supplementary Fig. 4a and Supplementary Table 9), thus providing protein-level evidence of their presence in human heart tissue. Furthermore, the protein abundance of these proteins is very similar between left ventricles of human and murine hearts (Pearson’s *r* = 0.85, Supplementary Table 10). To focus our analysis on proteins expressed in cardiomyocytes, we cross-checked our data against an scRNA-seq resource of the human heart published by Tucker et al.^34^. This analysis showed that 98% of the proteins in our network were expressed in at least one of the human cardiomyocyte subpopulations (Fig. 7c, Supplementary Fig. 4b and Supplementary Tables 9 and 11), with the main contribution coming from ventricular cardiomyocytes followed by atrial cardiomyocytes (Supplementary Table 12). Finally, we evaluated how cell type specific the interactomes are. We found a very high correlation of RNA expression of the interactors between different cardiomyocyte subpopulations (Pearson’s *r* > 0.9) and a drastically lower correlation when evaluating non-myocyte cell types (Supplementary Table 13).

Having confirmed human heart protein expression followed by expression specifically in cardiomyocytes, we next applied the genetic ECG resource across the entire network (Fig. 7d). For each protein interactor in the network, we queried the GWAS data across all timepoints in the ECG cycle to determine the most significant variant within the linkage-disequilibrium block of the corresponding gene. Combined, the results show that 340 of the proteins in the network were encoded in genetic loci that significantly associate with an ECG phenotype (Fig. 7e). This translates to 44% of the proteins in the ion channel interactome influencing human heart ECG.

## Discussion

Our modern understanding of cell electrophysiology is founded on the studies of Hodgin, Huxley and Katz, who first recorded electrical membrane currents from the squid giant axon^35^. The authors speculated those currents resulted from the flux of ions through ion-selective channel proteins whose identity, at that time, was unknown. Decades later, identification of genes encoding pore-forming proteins gave molecular identity to these currents. Yet, it was soon realized that though the currents flow through specific pore-forming proteins, the amplitude, time and voltage dependence of these currents are dictated by a complex network of molecular interactors^1,6,11,36^. Multiple studies have taken a one-molecule-at-a-time approach to build a catalog of these interactors, while more recent ones have applied high-throughput methods of data acquisition and analysis to characterize the interactors of a specific channel^37,38^. Here we combined proteomics and bioinformatics approaches to define in parallel the interactome of 13 cardiac ion channels, identify points of convergence between individual networks and examine the possible impact of the interactors on the electrophysiology of the heart.

Our approach was based on IP of individual ion channel proteins from cardiac membrane fractions. As an initial step, we tested a large panel of antibodies before selecting those that specifically recognized the protein of interest as bait. We then applied stringent criteria to determine whether a protein molecule detected in the precipitate should be considered as part of the interactome of that bait (described in Methods). Because of this stringency, weak yet functionally relevant dynamic interactors (for example, kinases) may have fallen below the threshold of inclusion. Furthermore, proteins that were detected in most precipitates were considered artifactual, although some may be true interactors. Despite such challenges, and to our knowledge, our study represents the most comprehensive compilation of the cardiac ion channel interactomes so far.

To validate some of the identified interactors functionally, we prioritized a set of candidates, ten in total, based on the extent to which they were enriched in the interactome of a given bait and, separately, on the fact that they were found to interact with multiple ion channels. For the functional evaluation on cardiac electrical properties, we performed high-resolution optical voltage mapping in zebrafish hearts where the respective interactors were acutely knocked out. Our results showed that loss of expression of the interactors we shortlisted for functional studies affected the overall electrophysiology of the heart, and in ways that were consistent with the roles of their ion channel partners. We characterized the longer-term effects of loss-of-function for three of the prioritized interactors by assessing changes to the ECG in adult KO fish compared to their WT siblings. After 8–12 months of possible compensatory remodeling, these fish still had evident disturbances in their electrophysiology. Intriguingly, of the ten interaction partners prioritized for functional studies, three were candidate genes near loci with genome wide significance in GWAS of atrial fibrillation (*MYZAP* (*P* = 2 × 10^−10^) (ref. ^39^), *SYNPO2L* (*P* = 9 × 10^−35^) (ref. ^39^) and *NEBL* (*P* = 2 × 10^−14^) (ref. ^40^)).

Our data showed that several cardiac ion channels have shared networks of interaction partners. Recently, De Smet et al. used two-color super-resolution imaging of adult cardiomyocytes to show that Gja1 (Cx43) and Cacna1c (Ca_V_1.2) co-localize at the cell end, and that intracellular calcium is a key regulator of the open state of Cx43 hemichannels^31^. Consistent with this, we found Cacna1c in the Gja1 interactome, and vice versa, and we also found Pln, an essential regulator of intracellular calcium homeostasis^41^, in the same network. Our data add a fundamental new observation, namely that channel proteins involved in repolarization are also a part of this hub. In particular, we observed the co-precipitation, and co-localization, of Gja1 (Cx43) with Kcnq1. It is worth noting that one of the shared proteins in the Kcnq1/Gja1 interactome is Nos1ap, a protein whose gene locus is highly associated with QT interval and sudden cardiac death in GWA studies^42–44^, though for molecular reasons yet to be understood. Kapoor et al. showed that Nos1ap is localized to the intercalated disc^45^, an observation consistent with the proteomics data presented here. Yet, the mechanism underlying the importance of Nos1ap in ECG parameters and in arrhythmogenesis remains to be fully determined^44,46^. Furthermore, both Kcnq1 and Gja1 co-precipitated ZO-1, a protein preferentially localized to the intercalated disc. The identification of Kcnq1 in the intercalated disc (among other locations) adds to studies showing Scn5a (Na_V_1.5) (ref. ^47^) and also calcium dyads in this domain^31^ and supports the idea that the intercalated disc functions as an organelle where major electrophysiological functions are represented.

For the outlining of the protein networks, we applied strict filtering criteria in the data analysis, and accordingly we may have lost transient interactors such as kinases and phosphatases. Likewise, interactions susceptible to the lysis conditions may have been lost, whereas certain abundant proteins may solely be present due to their higher abundance. Also, the protein interactions described here may occur at any point during the life cycle of the bait. In our reported networks, we do observe interactors that are involved in protein synthesis as well as protein degradation. In this regard, it is worth noting that the protein Naca is detected in the shared interactome of Kcnq1 and Kcnh2. Naca acts as a triage factor of nascent proteins to prevent their mislocalization following synthesis and facilitates their proper translocation to the correct subcellular domain^48^. The fact that Kcnq1 and Kcnh2 share this interactor and also co-localize at the membrane^30^ is consistent with recent findings of co-translation (and likely co-transport) of ion channel proteins encoded by different, but functionally related, genes^49^. This highlights the fact that protein–protein interactors operate not only at the site where the bait function as channels, but at multiple steps during synthesis, trafficking and degradation.

It should be noted that Kcnq1 is not a major component of action potential repolarization in the mouse heart^50^, though both transcript and protein can be detected and recessive phenotypes for *KCNQ1* in human are recapitulated. One possible explanation is that, in the murine heart, an interactor of Kcnq1 necessary for its function as a charge carrier is not expressed^51^. Additional experiments will be necessary to document the cardiac interactome of Kcnq1 in species where this outward current is manifest and to compare it to the interactome presented in the present study. Indeed, some channel interactors impact function in different ways depending on the model system, be it across species, across hearts and for some even across regions of the same cell. The calcium/calmodulin-dependent serine protein kinase, Cask, is reported as a direct partner of Na_V_1.5, but the functional effect of the interaction is restricted to channels localized to the lateral membrane^52^. Similarly, nanodomain-specific pharmacological modulation of Na_V_1.5 in cardiac myocytes has been demonstrated^53^, complementing findings that the steady-state inactivation of Na_V_1.5 is different in the cell end than in the mid-section of the cell^54^. This is likely to be consequent to the subcellular distribution of interactors and their effect on the gating properties of the channel. Other examples of differences in functional effects across model systems count overexpression of the Na_V_1.5 interactor LITAF (lipopolysaccharide-induced tumor necrosis factor-α factor) that substantially increase sodium current density in cardiomyocytes, while KO of the corresponding gene in zebrafish does not show a notable effect^55^, as well as general ionic current differences in cardiomyocytes across stages of maturity (for example, refs. ^56^ and ^57^). These and other examples indicate that there can be differences in the effect of interactors as a function of species, but also as a function of the regions of the heart or regions of the cell. In our study, we found divergent functional outcomes for the Na_V_1.5 interactor Glipr2 depending on the model system we evaluated it in. The concept that interactors can impact function in different ways, depending on the cell, the organism or the nanodomain in which they are tested underscores the need for additional functional studies to outline the ionic current mechanisms. In our study, the results consistently showed that loss of an interactor can affect the electrical phenotype, but there are some inconsistencies between the observations in mature (adult mammalian) myocytes and immature (fetal, or embryonic) cell systems. The reason remains unknown, though we speculate that it may have to do with the state of maturity of the myocyte.

To evaluate the relevance of our findings in the context of human heart electrophysiology we undertook a multi-omics data integration approach. We confirmed that more than 92% of the ion channel interactors are also expressed at the protein level in human hearts, and we report that the protein abundances of the channel interactors are similar in human and murine left ventricles. Due to the experimental design, our data does not reveal the cellular identity where the interactions outlined take place. However, we do show that the interactors are primarily expressed in cardiomyocytes, which are also the primary cell type expressing the ion channel proteins used as baits. The fact that the orthologous transcripts are primarily expressed in human cardiomyocytes, and that the protein orthologs are also present in human hearts, supports that the reported interactomes could be relevant to human cardiac electrophysiology. Using information from the UK Biobank, we implemented a strategy to characterize the potential role of a specific interactor on cardiac electrophysiology. Utilizing an ECG-GWAS tool developed by Verweij et al.^33^, we show that 44% of the interactors are encoded by genes in the vicinity of single-nucleotide polymorphisms significantly associated with variations in the ECG. This approach does not establish a physical association between a channel and a protein (our IP-based proteomics does that) but it does suggest that the interactor (or its region within the genome) may play a role in determining the overall electrical homeostasis of the heart.

Understanding the mechanisms of how each of the physical interactions documented herein may translate into functions affecting electrical homeostasis remains a matter for future studies. Herein, we have documented how two of the interaction partners contribute to electrical homeostasis by affecting the sodium current in adult cardiomyocytes. We report that silencing of the *Gsn* gene encoding for gelsolin led to increased sodium current density and affected the sodium channel kinetics by left-shifting the voltage dependency of channel activation in adult cardiomyocytes, and we confirmed close proximity of gelsolin and Scn5a in adult cardiomyocytes. Gelsolin is a Ca^2+^-dependent actin-binding protein that regulates actin filament assembly and disassembly. We speculate that the effect observed on sodium channel function in absence of gelsolin is coupled to actin microfilament rearrangements. Similar effects of gelsolin have previously been reported for non-voltage-gated sodium channels in leukemia cells^58^. Similar to the effect of gelsolin, we report that silencing of the gene *Epn2* leads to increased sodium current density in adult cardiomyocytes. *Epn2* encodes the protein epsin-2, which is involved in protein trafficking and specifically engage in trafficking of proteins via clathrin-coated vesicles^36^. In our study, absence of epsin-2 exclusively affected the sodium current density, but none of the kinetic parameters evaluated. Accordingly, we speculate that epsin-2 is involved in the trafficking of sodium channels in adult cardiomyocytes. Further specialized studies are required to fully understand the mechanisms of epsin-2 and gelsolin regulation of the cardiac sodium current.

In summary, we have utilized an array of experimental and bioinformatics methods to compile and analyze a comprehensive interactome of 13 cardiac ion channels isolated from murine heart tissue. The overall dataset includes known interactors and a multitude of new ones. Almost all identified channel interactors are also present in human cardiac tissue and predominantly so in cardiomyocytes. Bioinformatics evidence suggests that close to half of the interactors influence the human heart ECG. We functionally characterized the cardiac electrophysiological impact of ten of the identified interactors. The outcome of these experiments underscores the rich source of information contained in the dataset as a whole and presents a validation of the functional relevance of the reported interactions for the electrical homeostasis of the heart.

## Methods

### Tissue preparation and IP

All procedures were performed according to the European Union legislation for the protection of animals used for scientific experiments. Mice were housed in individually ventilated cage-systems with 8–10 air changes per hour, temperature: 22 °C (±2 °C), humidity 55% (±10%) and standard 12:12 h light:dark cycle. Food and water were provided ad libitum. Cardiac tissue homogenates were prepared from the hearts of 20 8-week-old male mice (C57BL/6JRj). The lysis and IP conditions such as detergent, input material and amount, and detergent type were optimized using Kcnq1 antibody (Alomone APC-022, Supplementary Table 1). The final experiments were carried out using 3 mg membrane enriched input material with the use of 1% NP-40, 1% sodium deoxycholate as detergents. This condition preserved the protein–protein interactions and gave minimal interference from blood proteins and better sequence coverage for the bait (Supplementary Table 1). In brief, hearts were dissected out and immediately snap frozen. The hearts of each mouse were homogenized separately using Precellys 24 (Bertin Instruments) in an ice-cold lysis buffer (50 mM Tris pH 8.5, 5 mM ethylenediaminetetraacetic acid, 150 mM NaCl and 10 mM KCl) supplemented with protease and phosphatase inhibitors (complete protease inhibitor cocktail (Roche), 1 μg ml^−1^ leupeptin, aprotinin and pepstatin, 1 mM phenylmethylsulfonyl fluoride, 5 mM sodium fluoride, 5 mM β-glycerophosphate and 1 mM sodium orthovanadate). The lysate was spun at 16,000*g* at 4 °C for 1 h to separate the membrane and the soluble fraction. The pellet containing the membrane fraction was re-solubilized in ice-cold lysis buffer supplemented with 1% NP-40, 1% sodium deoxycholate for 16 h at 4 °C. Subsequently, membrane fractions were centrifuged to remove debris, and membrane-enriched lysates from five mice were pooled together to obtain four replicates. The protein concentrations were determined using Quick Start Bradford Dye Reagent (Bio-Rad). IPs were performed by pre-clearing 3 mg of membrane fraction lysate per antibody using Dynabeads Protein G (Thermo Fisher). The pre-cleared lysate was incubated with 2 µg antibody (Supplementary Table 1) overnight at 4 °C. The complex was captured using Dynabeads Protein G, washed three times with lysis buffer, and eluted in 1× lithium dodecyl sulfate loading dye (Invitrogen) supplemented with 100 mM dithiothreitol by heating it at 70 °C for 10 min.

The reverse pulldowns of Inf2 were carried out in a similar manner as described above with the exception of using total tissue lysate without membrane enrichment. Briefly, the homogenates for triplicate experiments were prepared from heart of one 8-week-old male mouse (C57BL/6JRj) by solubilizing it in lysis buffer supplemented with detergents as mentioned above. IP was carried out using Anti-Inf2 antibody (Bethyl Labs, A303-428A), and the whole eluate was loaded on SDS–PAGE gels.

### MS sample preparation

The eluate was run on SDS–PAGE, and each of the lanes was excised into four parts, combining the IgG bands in one part. Each of the gel slices was in-gel digested with trypsin, as previously described^59^. Briefly, excised gel bands from Coomassie-stained SDS–PAGE-separated proteins were minced, destained, reduced and alkylated. The proteins were extracted and digested with sequencing-grade trypsin (Promega) overnight at 37 °C. The activity of trypsin was subsequently quenched by trifluoroacetic acid (TFA) acidification, and the resulting peptides were extracted by acetonitrile (ACN)/water and desalted and concentrated on C_18_ STAGE tips. Peptides were eluted with 2× 20 µl 40% ACN, 0.5% acetic acid, and the organic solvents were removed in a vacuum centrifuge.

For whole proteome measurements, the input membrane fraction was subjected to acetone precipitation to remove the detergents and the precipitated proteins were dissolved in 6 M GdnHCl. The proteins were reduced and alkylated followed by in-solution digestion with Lys-C (1:300 w/w) for 1 h at 37 °C, diluted 1:12 with 50 mM Tris–HCl (pH 8) to lower the GdnHCl concentration to 0.5 M. The peptides were further digested with trypsin (1:100 w/w) overnight at 37 °C. Reactions were quenched by addition of TFA, and samples were desalted on C_18_ SepPak columns (Waters). Then 100 µg peptide from each sample was used for fractionation on a Dionex UltiMate 3000 UPLC system (Thermo Scientific) into 12 concatenated fractions for in-depth proteome measurements. The organic solvent was removed using vacuum centrifugation.

### MS measurements and analysis

Peptide samples were analyzed by online reversed-phase liquid chromatography coupled to a Q-Exactive HF-X quadrupole Orbitrap tandem mass spectrometer (LC–MS/MS, Thermo Electron). Peptide samples were resuspended in 5% ACN, 0.1% TFA in 96-well microtiter plates and autosampled (2 µl injection volume) into a nanoflow Easy-nLC system (Proxeon Biosystems). Peptide samples were separated on a 15 cm self-pack 75 µm internal diameter PicoFrit columns filled with ReproSil-Pur C_18_-AQ 1.9 μm resin (Dr. Maisch GmbH). A 30 min multi-step linear gradient (Buffer A: 0.1% formic acid, Buffer B: 0.1% formic acid, 80% ACN) was used that went from 10% to 30% Buffer B in 25 min, 30% to 45% Buffer B in 5 min and 45% to 80% Buffer B in 30 s. Column effluent was directly ionized in a nano-electrospray ionization source operated in positive ionization mode and electrosprayed into the mass spectrometer. Full-MS spectra (350–1,400 *m*/*z*) were acquired after accumulation of 3 × 10^6^ ions in the Orbitrap (maximum fill time of 45 ms) at a resolution of 60,000. A data-dependent Top12 method then sequentially isolated the most intense precursor ions for higher-energy collisional dissociation. MS/MS spectra of fragment ions were recorded at a resolution of 15,000 after accumulation of 1 × 10^5^ ions in the Orbitrap (maximum fill time of 22 ms). Raw MS data were processed using the MaxQuant version 1.6.2.3 (ref. ^60^), and proteins were identified with the built-in Andromeda search engine by searching MS/MS spectra against an in silico tryptic digest of a database containing all reviewed SwissProt mouse protein entries. The MS/MS spectra were searched with carbamidomethyl-cysteine as fixed modification, as well as methionine oxidation, acetylation of protein N-termini and Gln→pyro-Glu and phospho(STY) as variable modifications. A maximum of two missed cleavages and six variable modifications were allowed. The minimum peptide length was set to 7 amino acids (default) and minimum Andromeda score required for modified peptides was 25, with minimum delta score of 6 (default). First search tolerance was 20 ppm (default) and main search tolerance was 4.5 ppm (default), requiring strict specificity of tryptic peptides. The match-between-runs option was enabled with default parameters for all runs of one bait protein. False-discovery rate cutoffs were set to 1% on peptide, protein and site decoy level (default), only allowing high-quality identifications to pass. All peptides were used for protein quantification.

### IP data analysis

The raw intensity measurements for the pulldowns and proteomes were quantile normalized separately using Bioconductor R package LIMMA^61^. Protein identification results were further processed using the Perseus software suite version 1.6.2.1 (ref. ^62^). All the proteins that were found in more than half of the pulldowns were used for median calculation, that is, 16 pulldowns will result in 64 replicates, and if a protein was found in more than 32 replicates it will be used further. Complementary median values were calculated for these proteins using the normalized values from other pulldowns. For pulldowns that belonged to the same family of proteins, such as Kcnj2, Kcnj3 and Kcnj5, the whole family was excluded from complementary median calculation. For the remaining half of the proteins without a median value, measured intensities from the control IgG pulldowns were used. If there were missing values still remaining, we first imputed values from the proteome measurements that were scaled to match those of the pulldowns using the method described in ref. ^21^ and then by random sampling from a left-shifted normal distribution according to default parameters in Perseus^62^.

Volcano plot visualizations were made in Perseus. We filtered the data to include only proteins found in at least three of the four replicates. The claim to significance was based on false discovery rate (FDR) of a two-sided *t*-test and s0 value (s0 controls the relative importance of *t*-test-based *P* value and difference between means)^62^. For most of the channels (Cacna1c, Gja1, Kcnd2, Kcnj2, Kcnma1, Kcnn3 and Scn5a) significance was claimed at FDR >0.05 and s0 >2. For the other channels, these cutoffs were tweaked to obtain the significant proteins. For Hcn4, Kcna5 and Kcnj5, we had to decrease the strictness which we believe was either due to the bait itself being low-abundant in cardiac tissue and/or the antibody efficacy and specificity. For Kcnh2, Kcnj3 and Kcnq1, we had to increase the strictness of claiming significance, which we believe is due to bait abundance or presence of bait in different micro-environments and organelles.

Despite the strict criteria for claiming significance, we obtained ~880 interactors for the 13 ion channels. Hence, to narrow down on select group of interactors to follow up on, we ranked them by the following criteria and chose one or two proteins per bait that were the top-most hits:

1. Number of replicate experiments in which the interactor was identified by MS/MS.
2. Sequence coverage of the protein.
3. The number of peptides measured in the bait IP compared to the number of peptides measured for this protein in any other IP.

This information was combined with the information we obtained from shared interactor analysis (given in later section) to highlight key proteins within the interactome for follow-up studies. These proteins are shown as yellow dots in Fig. 2.

### Protein immunoblotting

IP elutions were separated on 4–12% gradient NuPage Bolt Bis-Tris gels (Thermofisher) using the XCell SureLock Mini-Cell Electrophoresis System (Thermofisher). Proteins were transferred to a nonfluorescent Immobilon polyvinylidene fluoride membrane (45 μM; Millipore) in Bolt transfer buffer (Thermofisher) using a minitransblot cell (Bio-Rad). After transfer, the membranes were blocked for 1 h at room temperature in a blocking buffer consisting of Odyssey blocking buffer (LI-COR Biosciences) diluted 1:1 with phosphate-buffered saline (PBS). The membranes were incubated overnight at 4 °C in a blocking buffer containing primary antibodies (1:1,000 dilution). After being washed with PBST, the membranes were incubated for 45 min with fluorescently conjugated secondary antibodies diluted in a blocking buffer (1:10,000). Bound antibody was detected by the Odyssey CLx Imaging System (LI-COR Biosciences) using 800 nm and 700 nm channels.

### Zebrafish maintenance

Zebrafish (*Danio rerio*) were maintained in a dedicated fish facility at 28.5 °C with a stable circulating system which continuously filters, treats (with ultraviolet light) and aerates the water. All animal experiments using zebrafish were performed according to the European Union legislation for protection of animals used for scientific experiments and was approved by the Danish National Animal Experiments Inspectorate (license 2021-15-0201-00811) or were approved by the Institutional Animal Care and Use Committee at Brigham and Women’s Hospital and Harvard Medical School.

### Generation of acute KO in zebrafish

For each gene studied, WT AB/Tuebingen (AB/Tu) zebrafish were crossed, and the resultant fertilized eggs from a single clutch were divided at random into two groups, each group containing males and females, to be injected with either (1) a solution containing multiple gRNAs targeting respective gene(s)/isoform(s), as well as Alt-R tracrRNA, and Alt-R S.p. HiFi Cas9 Nuclease V3 (KO group), or (2) tracrRNA and Cas9 alone (control group). These components (all from Integrated DNA Technologies), were prepared according to the manufacturer’s instructions and microinjected into single-cell embryos delivering a total of 2 fmol of gRNAs and 250 pg of Cas9 in 1 nl per embryo. gRNAs were designed using CHOPCHOP (https://chopchop.cbu.uib.no/)^63^. Sequences of the gRNAs and respective target(s) are listed in Supplementary Tables 3 and 4. The remains of all KO-targeted embryos used for voltage mapping were sequenced using the listed primer pairs. The efficiency of editing varied across gRNAs and respective target sites but ranged from 84% to 100% at two sites or more in each targeted embryo. Only embryos with evidence of gene edits were included in the KO group for the final analysis. A minimum *n* of 9 zebrafish embryos was used for these studies.

### Optical mapping of isolated zebrafish hearts

Optical mapping and signal processing were performed as previously described^27^. Briefly, isolated hearts from either 3 or 5 days post-fertilization embryos were incubated with a voltage-sensitive fluorescent dye in the FluoVolt Membrane Potential Kit (Invitrogen) for at least 15 min at room temperature. Afterwards, the hearts were transferred into a perfusion chamber (RC-49MFS; Warner Instruments) filled with normal Tyrode solution containing 1 mM cytochalasin D (Sigma) to decouples electrical impulses from contractions. The chamber was then mounted onto the stage of an inverted microscope (TW-2000; Nikon) with built-in platinum wires connected for field pacing. The fluorescent dye was excited with a 470 nm light-emitting diode, and the emission was collected by a high-speed 80-by-80-pixel charge-coupled device camera (RedShirtImaging) with 14-bit resolution. Using a 20× objective and 0.5× C-mount adapter, the final magnification was 10× with a pixel-to-pixel distance of 2.4 µm. For signal processing and quantification, images were analyzed by customized scripts in MATLAB version R2019a (MathWorks)^27^.

### Electrocardiography of adult zebrafish

Recordings were performed as previously described^64^. Briefly, adult (3–4 months) male and female zebrafish were anesthetized with 0.016% tricaine and maintained in a wet sponge. A sensing electrode was placed above the cardiac region, and a reference electrode was placed caudally in the sponge. The fish were recorded for at least 5 min, and roughly 30 consecutive beats used to construct a signal averaged ECG for analysis, all performed in LabChart 8 (ADInstruments). ECG characteristic parameters were tested for significant difference in magnitude between the acute CRISPR KOs and their control clutchmates by the two-sided Mann–Whitney *U* test. Where noted, parameters were also tested for significant difference in group variance between groups using the Conover squared-ranks test for homogeneity of variance.

### Design and delivery of AAV-shRNA for gene silencing in mice

To silence the gene expression of Epn2, Glipr2 and Gsn in mice, three vectors of pAAV9[shRNA]-EGFP-U6>mEpn2, mGlipr2 and mGsn were designed. AAV-shRNA vectors were generated and produced by VectorBuilder in the titer 2 × 10^13^ GC ml^−1^. The shRNA target sequences for each gene were: mEpn2[shRNA] CAGTGGCTCCTTCGAACATTA; mGlipr2[shRNA] CAGCCATGGTATGGAAGAATA; mGsn[shRNA] GACTTCTGCTAAGCGGTACAT. AAV-EGFP-shRNA for sodium channel protein interactors and the empty vector expressing GFP only were diluted in PBS administered via the tail vein (injection volume between 80 and 100 µl) in doses of 4 × 10^13^ vg kg^−1^. Mice with no AAV injections were also included as controls to ensure that AAV injection alone did not affect electrophysiological properties. The C57BL/6 mice were from Charles River Laboratories and 23 between injected and not injected 3–4-month-old female mice were used for AAV-shRNA gene silencing. Temperature (21–23 °C) and relative humidity (30–70%) were maintained according to standard protocols put in place by the Division of Comparative Medicine at New York University (NYU) Grossman School of Medicine. Lighting was provided via an automatic timer with a 12 h light–dark cycle.

Animals were treated in accordance with the Guide for Care and Use of Laboratory Animals published by the US National Institutes of Health. Procedures were approved by the NYU Institutional Animal Care and Use Committee (IACUC) committee under protocol number 160726-03.

### Cardiomyocyte dissociation

Murine ventricular myocytes were obtained by enzymatic dissociation 17–21 days after AAV infection, following standard procedures. Briefly, mice were injected with 0.2 ml heparin (500 IU ml^−1^ intraperitoneally) 20 min before heart excision and anesthetized by inhalation of 100% CO_2_. Deep anesthesia was confirmed by lack of response to otherwise painful stimuli and cervical dislocation were performed after anesthesia to ensures the death of the animal. Hearts were quickly removed from the chest and placed in a Langendorff column. For cell dissociation, the isolated hearts were perfused sequentially at a constant flow rate of 3 ml min^−1^ with solution containing (in mmol l^−1^): 113 NaCl, 4.7 KCl, 1.2 MgSO_4_, 0.6 Na_2_HPO_4_, 0.6 KH_2_PO_4_, 12 NaHCO_3_, 10 KHCO_3_, 10 HEPES, 5.5 glucose and 30 taurine, pH 7.40 with NaOH and then an enzyme (collagenase type II 600 U ml^−1^, Worthington) solution with the addition of low CaCl_2_ concentration (12.5 µM) for 15 min. Perfusate temperature was maintained at 37 °C. After digestion, ventricles were separated into small pieces and gently minced with a Pasteur pipette. The isolated cardiomyocytes were suspended in stop buffer (low-calcium solution with 5% bovine calf serum), and the Ca^2+^ concentration was increased gradually to normal values. Cardiomyocytes were used on the same day of isolation.

### Patch-clamp measurements from adult murine cardiomyocytes

Only the eGFP.positive cardiomyocytes were used to perform the sodium current recordings. All whole-cell *I*_Na_ recordings were conducted at room temperature using an Axon multiclamp 700B Amplifier and a pClamp system (versions 10.2, Axon Instruments). Pipette resistance was maintained within the range of 1.8–2.2 MΩ. Recording pipettes were filled with a solution containing (in mmol l^−1^): NaCl 1, CsF 135, EGTA 10, Na_2_ATP 5 and HEPES 5, pH 7.2 with CsOH. Cells were maintained in a solution containing (in mM): NaCl 5, CsCl 130, CaCl_2_ 1, MgCl_2_ 1, CdCl_2_ 0.1, HEPES 20, TEACl 10 and glucose 5, pH 7.35 with NaOH. To determine the peak current voltage relation, 300 ms voltage pulses were applied to *V*_m_ −90 mV to 0 mV in 5 mV voltage steps, from a holding potential of *V*_m_ = −140 mV. Interval between voltage steps was 1.7 s. Current densities were determined by dividing current amplitude by the cell capacitance (*C*_m_), as determined by application of +20 mV depolarizing test pulses. Steady-state activation curve was calculated using the equation: *I* = gmax × (*E* − *E*_rev_), where ‘*I*’ is the peak current amplitude; ‘gmax’ is the maximal conductance; ‘*E*’ is the voltage applied; ‘*E*_rev_’ is the Na^+^ reversal potential, calculated for each cell. Steady-state inactivation curve was determined by stepping *V*_m_ from −130 mV to −40 mV, followed by a 30 ms test pulse to *V*_m_ = −30 mV to elicit *I*_Na_. The steady-state voltage-dependent inactivation and activation curves were fitted to Boltzmann’s functions.

### Real-time PCR evaluation of Epn2, Gsn and Glipr cardiomyocyte knockdown

All cardiomyocytes remaining after patch clamp measurements were washed in PBS and utilized for RT–PCR measurement. The gene transcripts of msEpn2, Glipr2 and Gsn in AAV9-shRNA injected mouse hearts were detected with real-time PCR techniques in the respective knockdowns. Total RNA was extracted and purified using RNeasy Mini Kit (QIAGEN) from the mouse cardiomyocytes. Complementary DNA was generated using High Capacity Reverse Transcript Kit (ThermoFisher) with equal amount of total RNA from silenced and control cardiomyocytes. Real-time PCR was performed with PowerUp SYBR Green reagents and applied on StepOnePlus Real Time PCR System. Relative quantification was calculated as RQ = 2^−ΔΔCT^. The RQs of each reaction were normalized to control mice injected with empty AAV-EGFP vector. The data were analyzed using GraphPad Prism 9.3.1 software, and the *P* values were calculated by *t*-test. The RNA samples were obtained from five AAV-shRNA-injected mice, and for each gene GAPDH was used as reference gene with forward primer: TGACGTGCCGCCTGGAGAAA and reverse: AGTGTAGCCCAAGATGCCCTTCAG; mouse Epn2 forward primer: GGGCAAGTGACACTGCTATAA and reverse: GAGGCTATCCTAGACCCTTTCT; mouse Glipr2 forward primer: GATAGGTGGTACAGCGAAATCA and reverse: CCACGCCTATCTTCTTGGTATT; mouse Gsn forward primer: ACTGTGCAACTGGATGACTAC and reverse: CCACACCTCCTTTCTTGTACTT.

### Cardiomyocyte dissociation for STORM imaging

Adult mouse ventricular myocytes were obtained by enzymatic dissociation. All procedures conformed to the Guide for Care and Use of Laboratory Animals of the National Institutes of Health and were approved by the NYU IACUC committee. Temperature (21–23 °C) and relative humidity (30–70%) were maintained according to standard protocols put in place by the Division of Comparative Medicine at NYU Grossman School of Medicine. Lighting was provided via an automatic timer with a 12 h light–dark cycle. The C57BL/6N mice were from Charles River Laboratories and 3–4-month-old male and female mice (ten in total) were injected with 0.1 ml heparin (500 IU ml^−1^ intraperitoneally) before heart excision and anesthetized by inhalation of 100% CO_2_. Deep anesthesia was confirmed by lack of response to otherwise painful stimuli. The mouse was then killed by cervical dislocation and the heart surgically removed via thoracotomy and placed in a Langendorff column. The isolated hearts were perfused sequentially at a constant flow rate of 3 ml min^−1^ with Ca^2+^-free solution containing (in mmol l^−1^): 113 NaCl, 4.7 KCl, 1.2 MgSO_4_, 0.6 Na_2_HPO_4_, 0.6 KH_2_PO_4_, 12 NaHCO_3_, 10 KHCO_3_, 10 HEPES, 5.5 glucose and 30 taurine, pH 7.40 with NaOH and then an enzyme (collagenase type II; Worthington) solution for 10 min. The temperature of the perfusion buffers was maintained at 37 °C. After digestion, the tissue was cut into small pieces, and minced by gentle mechanical agitation with a Pasteur pipette. The isolated cardiomyocytes were suspended in 10 ml of stop buffer (Ca^2+^-free perfusion buffer with 5% bovine calf serum) and the Ca^2+^ concentration was increased gradually to 1.0 mmol l^−1^. Cardiomyocytes were kept in Tyrode’s solution containing (in mmol l^−1^): 148 NaCl, 5.4 KCl, 1.0 MgCl_2_, 1.0 CaCl_2_, 0.4 NaH_2_PO_4_, 15 HEPES and 5.5 glucose, pH 7.40.

### Single-molecule localization microscopy by STORM

Freshly isolated ventricular cardiomyocytes from WT mice were plated on laminin-coated coverslips and fixated in 4% paraformaldehyde (PFA)–PBS. Cells were then permeabilized with 0.1% Triton X-100 in PBS for 10 min. Blocking was done in PBS containing 2% bovine serum albumin (Sigma), 2% glycine (Sigma) and 0.2% gelatin (Sigma) for 30 min. Primary antibodies diluted in blocking buffer were incubated for 1 h at room temperature, followed by three washes in PBS, and secondary antibodies incubation for 15 min. Primary antibodies used were: rabbit polyclonal Kcnq1 (1:50, Alomone catalog number #APC-022), rabbit polyclonal Kcnq1 (1:50, Alomone catalog number #APC-168), mouse Anti-Connexin 43 clone 4E6.2 (1.50, Sigma-Aldrich catalog number #MAB3067, Lot: 3138211), rabbit polyclonal Scn5a (1:50, Sigma catalog number #S0819, Lot: SLBW8952), rabbit monoclonal anti-Gelsolin clone EPR1941Y conjugated to Alexa Fluor 647 (1:100, Abcam catalog number #ab75832), mouse anti-α-actinin clone EA-53 conjugated to Alexa Fluor 488 (1.300, Sigma-Aldrich catalog number #A7811, Lot: 0000141496)), Alexa Fluor goat anti-rabbit 568 (1:10,000, Invitrogen, catalog number #A11011, Lot: 1778925), Alexa Fluor goat anti-rabbit 647 (1:10,000, Invitrogen, catalog number #A21244, Lot: 1834794) and Alexa Fluor goat anti-mouse 488 (1:10,000, Invitrogen, catalog number #A11001, Lot: 2220848).

Samples were imaged using a custom-built platform based on an inverse microscopy setup Leica DMI3000 as described before^65^. Imaging conditions were achieved by addition of 200 mmol l^−1^ mercaptoethylamine and an oxygen scavenging system (0.4 mg ml^−1^ glucose oxidase, 0.8 µg ml^−1^ catalase and 10% (wt/wt) glucose) to the fluorophore-containing sample.

Movies containing 2,000 frames were submitted to a home-built software in MATLAB for precise single-molecule localization. Reconstructed super-resolved images were processed with a smoothing filter (‘Gaussian blur’ function in ImageJ), adjusted for brightness and contrast, and filtered to a threshold to obtain a binary image. Cluster detection and parameters were obtained using the function ‘Analyze particles’ in ImageJ version 1.53a.

As described previously^65^, to standardize measurements, distances between Scn5a and Gsn, or between Kcnq1 and Cx43, were measured with an automated script written in Python. The script utilized the image processing packages scikit-image, and ‘Mahotas’, version 1.2 an open-source software for scriptable computer vision^66^. Two clusters were considered separate if one was at least 20 nm/1 pixel apart from another in any direction.

### Protein–protein interaction networks

Protein–protein interaction networks were created in Cytoscape version 3.8.0.

### Comparison of interactor protein profiles in human and mouse hearts

To evaluate the correspondence between murine cardiac protein abundances and those of human heart, we turned to analyses of cardiac biopsy samples. Specifically, we have evaluated the protein abundance profile for the cardiac proteins we have identified. For each of these proteins we extracted the protein abundance in murine and human hearts across the three chambers available across these species from a study our group published last year^67^. This dataset is unique for such comparisons, because all samples have been processed and analyzed together and hence the dataset enables comparison across species. Additionally, we calculated Pearson correlation coefficients to determine the strength of correlation between protein expression levels of the interactors in the different chambers of within one species as well as between the same chambers between mouse and human hearts.

### Intersection with human proteomics data

To assess which of all interactors are also expressed in the human heart, we checked for each interactor if it was identified in our deep proteomics atlas of the human heart, which is based on more than 1,000 MS runs (unpublished data).

### Intersection with single-cell RNA expression data

Similarly, to evaluate which of all interactors are expressed in human cardiomyocytes, we queried a publicly available scRNA-seq dataset of 287,269 cells of the human heart via the Single Cell Portal and extracted the mean expression values per cell type of all interactors for each bait protein^34^. For each bait protein, as well as for all combined, we then calculated the percentage of interactors that are expressed in cardiomyocytes, that is, have a non-zero relative expression value. Additionally, we calculated Pearson correlation coefficients to determine the strength of correlation between RNA expression levels of the interactors in different cardiac cell populations. Finally, we calculated the summed mean expression of all interactors per cell type to determine from which cell type(s) the identified interactors are likely to originate.

### Previously known interactors

To determine which of all identified interactors were previously known interactors of their bait protein we combined data from three different public resources, namely STRING^23^, Bioplex^22^ and InWeb^15^. From STRING we restricted the search to interactors based on experiments and databases with a minimum interaction score of 0.4 to exclude interactors with low confidence.

### ECG plotter analysis and resting heart rate GWAS

Mouse gene names were converted to their human orthologs using Ensembl biomart ortholog mapping database (downloaded 24 November 2020). In case a gene was not found in the ortholog database the mouse gene symbol was converted to all capital letters.

ECG plotter was used to determine if there is a significant influence of each interactor on any time windows of the ECG cycle^33^. For this, we adapted the original ECG plotter script and determined the LD block of each gene using the LDlink database before testing the influence on the ECG of all single-nucleotide polymorphisms in the results LD block^68^.

To correct for multiple hypothesis testing, we used the number of independent tests for the 500 datapoints of the ECG profile as determined in the ECG plotter publication (that is, 80) and the number of proteins in the network that were tested. In case of the 10 proteins selected for follow-up experiments this resulted in a significance cutoff of 0.05/80/10 = 6.25 × 10^−5^ and in case of the whole interactome network in 0.05/80/881 = 7.09 × 10^−7^.

### Reporting summary

Further information on research design is available in the Nature Portfolio Reporting Summary linked to this article.

### Supplementary information

Supplementary Information Supplementary Tables 1–13 and Figs. 1–4.

Reporting Summary

Supplementary Tables Supplementary Tables 6, 8, 9 and 11.

### Source data

Source Data Fig. 1 Statistical source data.

Source Data Fig. 2 Statistical source data.

Source Data Fig. 3 Statistical source data.

Source Data Fig. 4 Statistical source data.

Source Data Fig. 5 Statistical source data.

Source Data Fig. 6 Statistical source data.

Source Data Fig. 7 Network source data.

Source Data Extended Data Fig./Table 1 Unprocessed western blots.

Source Data Extended Data Fig./Table 2 Statistical source data.

Source Data Extended Data Fig./Table 5 Statistical source data.

Source Data Extended Data Fig./Table 6 Statistical source data.

Source Data Extended Data Fig./Table 8 Statistical source data.

## Data Availability

The MS proteomics data have been deposited to the ProteomeXchange Consortium via the PRIDE partner repository with the dataset identifier PXD028021 and project name ‘Identification of cardiac ion channel protein networks and their signature in the human electrocardiogram’. Mouse protein sequence database was downloaded from https://www.uniprot.org/, reviewed sequences only. Human–mouse ortholog data were downloaded from Esembl BioMart: https://www.ensembl.org/info/data/biomart. Previously known interactors were extracted from BioPlex (https://bioplex.hms.harvard.edu/interactions.php (BioPlex 3.0 Interactions (293T cells))), STRING (https://string-db.org/) and InWeb (10.1038/nmeth.4083). The Tucker et al.^34^ single-nucleus RNA sequencing dataset was downloaded from Single Cell Portal (https://singlecell.broadinstitute.org/single_cell, dataset number SCP498). Human ECG GWAS data were downloaded from ECGGenetics (https://www.ecgenetics.org/). Source data are provided with this paper.

## Code availability

The R script for the ECG plotter analysis is provided in our GitHub repository https://github.com/CardiacProteomics/ion-channel-interactomes. The Python script for STORM imaging can be made available upon request.
