# Supplementary material for: Outlining cardiac ion channel protein interactors and their signature in the human electrocardiogram
Source: Nat Cardiovasc Res. 2023 Jul 13;2(7):673–92. doi: 10.1038/s44161-023-00294-y (PMC11041666; doi:10.1038/s44161-023-00294-y)
Supplement: Supplementary file 1 — Supplementary Tables 1–13 and Figs. 1–4. [file 44161_2023_294_MOESM1_ESM.pdf]

nature cardiovascular research

Article https://doi.org/10.1038/s44161-023-00294-y

Outlining cardiac ion channel protein interactors and their signature in the human electrocardiogram

In the format provided by the authors and unedited

nature cardiovascular research

Article https://doi.org/10.1038/s44161-023-00294-y

Outlining cardiac ion channel protein interactors and their signature in the human electrocardiogram

In the format provided by the authors and unedited

Supporting Information

Contents

Supplementary Tables 2

Supplementary Table 1: Overview of antibodies used and IP conditions 2

Supplementary Table 2: Number of proteins/significant interactors per bait experiment and cut-offs applied 4

Supplementary Table 3: gRNA for zebrafish acute knockout I. 5

Supplementary Table 4: gRNA for zebrafish acute knockout II. 6

Supplementary Table 5: Cardiac electrophysiology of zebrafish acute knockout I. 7

Supplementary Table 6: Cardiac electrophysiology of zebrafish acute knockout II (Excel file). 8

Supplementary Table 7: Repolarization abnormality in adult nrap knockout zebrafish. 8

Supplementary Table 8: Electrocardiogram parameters from adult knockout zebrafish (Excel file). 8

Supplementary Table 9: Table showing the data from protein interaction networks in Fig. 7 in table format (Excel file). 9

Supplementary Table 10: Comparison of protein expression in mouse and human hearts 9

Supplementary Table 11: Percentage of interactors expressed in cardiomyocytes (Excel file). 9

Supplementary Table 12: Summed mRNA expression of all interactors per cell type 10

Supplementary Table 13: Similarity of mRNA expression of interactors between cell types 10

Supplementary Figures 11

Supplementary Figure 1 11

Supplementary Figure 2 12

Supplementary Figure 3 13

Supplementary Figure 4 14

Supplementary References 15

Supplementary Tables

Supplementary Table 1: Overview of antibodies used and IP conditions

Antibodies used for immunoprecipitations: The antibodies were used in a 1:1000 ratio for immunoblots and 2 µg antibody was used for immunoprecipitation experiments per replicate.

Sr. No.	Antibody cat #	Description
**1**	APC-022 (Alomone)	Anti K_V7.1
**2**	APC-062 (Alomone)	Anti K_V11.1
**3**	ACC-003 (Alomone)	Anti-Ca_V1.2
**4**	ASC-005 (Alomone)	Anti-Na_V1.5
**5**	APC-004 (Alomone)	Anti-K_V1.5
**6**	APC-023 (Alomone)	Anti-K_V4.2
**7**	APC-052 (Alomone)	Anti HCN4
**8**	APC-026 (Alomone)	Anti-K_{ir}2.1
**9**	APC-005 (Alomone)	Anti-K_{ir}3.1 (GIRK1)
**10**	APC-027 (Alomone)	Anti-K_{ir}3.4 (GIRK4)
**11**	APC-021 (Alomone)	Anti-K_{Ca}1.1 (1097-1196)
**12**	ACC-201 (Alomone)	Anti Cx43
**13**	APC-025 (Alomone)	Anti-Kcnn3
**14**	ab37415 (Abcam)	Control IgG Rabbit
**15**	A303-428A (Bethyl labs)	Anti-Inf2

Antibodies used for STORM imaging:

Sr. No.	Antibody cat #	Description
**16**	ab75832 (Abcam)	Anti-Gelsolin
**17**	A7811 (Sigma)	Anti-α-Actinin
**18**	S0819 (Sigma)	Anti-Na_V1.5
**19**	MAB3067 (Sigma)	Anti-Cx43

Evaluation of experimental conditions for immunoprecipitation using Kcnq1 APC-022 antibody:

**Detergent in lysis buffer**	**Source of input material**	**Amount of input (mg)**	**Total number proteins identified by LC-MS/MS**	**Number of blood proteins identified**	**Kcnq1 sequence coverage (%)**	**Number of known KCNQ1 interactors identified**	**Overall best condition**
1% NP-40/1% Sodium deoxycholate	Membrane-enriched	1	296	3	25.3	5	
		3	557	5	42.5	8	x
1% NP-40/1% Triton X-100		1	149	5	25.7	2	
		3	334	8	41.3	5	
1% Triton X-100		1	83	3	28.4	1	
		3	224	7	33.4	4	
1% NP-40/1% Sodium deoxycholate	Total cardiac lysate	1	291	3	25.1	3	
		3	441	5	33.1	4	
1% NP-40/1% Triton X-100		1	114	6	23.2	1	
		3	216	7	37.1	2	
1% Triton X-100		1	62	5	28.1	1	
		3	175	8	32.6	4	

Supplementary Table 2: Number of proteins/significant interactors per bait experiment and cut-offs applied

Bait	Total proteins	Significant interactors	S0 cut-off	FDR cut-off
Cacna1c	1024	156	2	0.05
Gja1	817	90	2	0.05
Hcn4	808	29	1	0.05
Kcna5	531	10	1	0.05
Kcnd2	983	117	2	0.05
Kcnh2	1247	229	3	0.01
Kcnj2	893	62	2	0.05
Kcnj3	1111	175	3	0.05
Kcnj5	804	46	1	0.05
Kcnma1	980	147	2	0.05
Kcnn3	833	41	2	0.05
Kcnq1	1488	366	3	0.005
Scn5a	611	60	2	0.05

Supplementary Table 3: gRNA for zebrafish acute knockout I.

Genes targeted, sequences of injected gRNAs, and sequencing primers used to confirm gene editing in the generation of acute knockout models in zebrafish.

Mouse gene	Zebrafish homolog(s)	gRNAs used	Exon targeted	Sequencing primers (forward; reverse)
Gsn	*gsna*	CACCCTGAGTTTGAGCGAGCGGG	2	GTGTTCATTCTGGGGTCATTTT; TTTCTATTTGTCCTGCAAACGA
		ACTGCACGCAAGATGAGAGTGGG	3	
	gsnb	GCATGTACAGTTGACGAGAGTGG	3	GGGTTCTACACGGGTGATACAT; TGACTCAAATCCCTGGACTTCT
		CCAACTCATGGGTACCTCAGTGG	4	AATCGTCTTTAGCAAGGTGGAG; TCACCTGTCCAAGGTTTAGGAT
Glipr2	*glipr2*	AGTGACCTGGAGAAACCACTAGG	5	GGATTTAGCTCCAAAACAGGTG; ccaatgacatctccacattgaa
		CTAGTTTAGAGCCAGTCGGCAGG	5	
	glipr2l	TACTGCTGAGTTTTAACGGTGGG	2	TGCTTCATTTTTGTTCATTTGG; TAAATGTGTGGGGTGACAGAAG
		CCGAGAGCCTTGCCAGCACCAGG	3	
Epn2	*epn2*	CAACTTCCAATGACCCATGGGGG	2	CAACAATTCGGAGACAAATGAA; TGGCAAGTTGACAAGCTAAAGA
		CAAAACGGGCTCAGAGCGAGTGG	2	
		TTTCTCTGACGTTGATGCCCTGG	2	
		GTACTCCTCCGAGTAACTGGTGG	2	
Inf2	*inf2*	ACCTGAGTAGTTCACCACAGAGG	3	acacaagccagcctaataaaaca; TTTAGCAACTGCTTGGTGAGAG
		AGCATCCGCGTCCTGACAGCCGG	3	
		CTGGACCGGCTGTCAGGACGCGG	3	
		ACTTGCCTTGTAGTGCTCCAGGG	4	CGTCCAACACTATGGTGAAAAA; TTTAGCAACTGCTTGGTGAGAG
Nebl	*nebl*	CAAAGGATGCTTCCATTGTGAGG	2	ACGCAACAGAGAAAGTGAACTG; AAAGGCTGTATGTGGAACCATC
		TCAACATACGCGTTGCAGTAGGG	2	
		GAAGTGATCTTACCTGACTTTGG	3	TAGTGGCAGACACTCCTGAAAA; TCACGATTTTCCCCTTAAAATG
		TACATTACTGATCTGATCCTGGG	4	CAGGAAGGATTTTGAACAGAGC; GTCCCTGCTCCAGTTTGAATAC
Nrap	*nrap*	CTTTGGATCTCATATCATAGTGG	4	GGTTCGGTTTCTGTATGTTTGC; TTGTGGTTACTCACCTGACTGG
		GGGACACACGTACAATCTCCTGG	5	CCAGTCAGGTGAGTAACCACAA; TGTAAAGCATTTGTTCCTCGAA
		ATCCTGGGGTGATCATGGCTGGG	7	TTGCACAAACTGTGTTTCAGTG; TGGCCTTTCTTGTACTCCAACT
		GATTACCCCTGGATACCAGATGG	11	AAGAAAACGCAGAGCACTTCAG; AATGACAGACCCCTCAAACAGT

Supplementary Table 4: gRNA for zebrafish acute knockout II.

Genes targeted, sequences of injected gRNAs, and flanking primers used to confirm gene editing in the generation of acute knockout models in zebrafish.

Human gene	Zebrafish orthologue(s)	gRNAs used	Exon	Flanking primers (forward & reverse)
PDE4DIP	pde4dip	GgAATGACCCGGGATGGCCG	1	AGAGTAAGGACGTGTGTCGGAT<br>AAATCGGTACATTCTGTCCAGC
		GgCTTGGTAGGATGCTAAGG	10	GTTGACCCAAGAAGACACACAA<br>gATGATTTCCTCTTTGACGCTC
SYNPO2L	synpo2la	GgTATCATCACCTTGTCGGG	1	AACATGGAGCGTGTGTACTGAG<br>CACATAAACCGACGGTACTGAA
		GGTCTCAGTGGAAGTTTGGG	3	GGATGCCCAAATGTACAAACTT<br>TCCTGAAACCCACTGTCTTCTT
	synpo2lb	GgGTGGCTATGAGAGCACAC	3	CCAGTACTTCTCCTGCCAAGAT<br>CTGCTTCTGCAGAGTACCCATT
		GgTCGGCTCCTCCTTGTAAT	1	GAAAATGGTAGCAGAGGAGGTG<br>CTCACAAACAAAAGCCAAACTG
NLRX1	nlrx1	gGCAATCATGGGAAGGCTCG	5	CTTATCCTAGAACGTCATCCCG<br>AGAGATCCATCACACCAGTCCT
		GGTGAACGAAGTCCGCTTGT	6	ACGCTTCATTTACTGCATTTCA<br>GAGCTCTTCGTCTGTTTTGGTT
MYZAP	myzap	gGGGCCCAGAGTCTATGGTG	4	GGTCTTCATGCACCTAAACACA<br>GATGTACCTCATCTCGTCCCTC
		GGAGTTTTCGGACTGTCGTC	2	ATCCTGCTGTGTGTTCATCTGT<br>CCAATCTGTCTGACTGCAAGAA
NEBL	nebl	gGAGGTGTCAGGTCTGCAAG	5	GTGTGGCGTCTCTCTTCTTTTT<br>GTACAGCCTTGTGTGTGCTGTT
		GgACCCTCATTGCGCGCGCTG	1	TCGGAGTTGTCGGAGAAGTTAT<br>AAGCATCCTTTGTGCCAATACT
EPN2	epn2	GgTCTGAGCCCGTTTTGATC	2	ACTTACAATGTGGTGGCCTTTT<br>TTTTCCATCCCGATCGATATAC
		GGACGACCTACGGCTCCAGA	4	AATCATCATGAATTGCAACAGC<br>CTTTTTCTTGGACACTTTGCCT
FYCO1	fyco1a	GgAGGAGAACTTGTGCAGAT	3	TTCTTACAGATGCTGTTTCCGA<br>TTAGGAGTCAGGTGCATGAAAA
		GGAGCTACAACGTAACCTGG	8	AGCAAAGAGAGCTTATTGACCG<br>TCCTGAAGGTCTTTATGGGAAA
	* fyco1b			
NRAP	nrap	GgGGGCTATGAACAGCAGAG	9	AAGGGAAGATCGTCAGTGATGT<br>CACCTGGTATCCTGGAGTGAAG
		GgAATCAGAGGCGGTCAGTG	3	CATATCTCACAGGCATAACCCA<br>GAAAAGGTGTATGAGGTTCTTGG

Efficient T7 in vitro transcription of gRNA requires guanine as the first two nucleotides; lowercase 'g' is used where this causes gRNA sequence to differ from zebrafish consensus sequence (GRCz11) of the CRISPR target. The terminal PAM sequence (NGG) of the target is not part of the gRNA sequence.
* fyco1b (Zfin:ZDB-GENE-130326-1) primary isoform (UniProtKB:F1RDW0) is missing several domains present in FYCO1 and was not targeted.

Supplementary Table 5: Cardiac electrophysiology of zebrafish acute knockout I.

Cardiac electrophysiology of zebrafish embryos (5 days post fertilization) with acute gene(s) knockout using multi-gRNA CRISPR/Cas9.

Cardiac channel	Gene	Ventricular CV (mm/s)		Atrial CV (mm/s)		Ventricular Vmax (1/s)		Atrial Vmax (1/s)		Ventricular APD_{80} (ms)		Atrial APD_{80} (ms)	
		control	KO	control	KO	control	KO	control	KO	control	KO	control	KO
Na_V	*gsna / gsnb*	31.7 ± 2.8	20.2 ± 2.7 *	3.3 ± 0.2	2.9 ± 0.2	84.4 ± 4.7	74.4 ± 3.6	46.0 ± 4.0	49.8 ± 3.2	346 ± 28	286 ± 17	252 ± 22	244 ± 14
Na_V	*glipr2 / glipr2l*	25.7 ± 4.5	12.6 ± 2.0 **	5.3 ± 0.4	3.8 ± 0.3*	81.8 ± 2.3	55.1 ± 3.4 ****	56.4 ± 3.8	39.6 ± 2.7 **	264 ± 15	456 ± 52 **	188 ± 15	231 ± 15
Na_V	*epn2*	24.5 ± 1.7	18.3 ± 1.8 *	5.9 ± 0.6	10.2 ± 2.2	94.2 ± 4.4	97.5 ± 4.2	40.5 ± 1.1	45.5 ± 2.0 *	241 ± 8	283 ± 14 *	128 ± 3	145 ± 8
Ca_V	*inf2*	33.7 ± 2.2	20.2 ± 1.4 ***	4.3 ± 0.6	4.6 ± 0.8	87.7 ± 3.9	84.0 ± 2.2	55.1 ± 2.4	47.0 ± 4.0	313 ± 20	332 ± 30	207 ± 11	248 ± 17
K_V	*nebl*	27.3 ± 3.1	27.2 ± 3.0	6.9 ± 0.4	4.2 ± 0.8*	94.7 ± 6.6	84.5 ± 3.7	59.4 ± 7.9	49.3 ± 3.8	269 ± 20	366 ± 30 *	224 ± 13	256 ± 28
K_V	*nrap*	26.3 ± 3.0	24.9 ± 3.4	4.1 ± 0.4	4.4 ± 0.6	85.7 ± 4.6	79.7 ± 6.5	50.1 ± 2.6	45.2 ± 4.1	304 ± 17	372 ± 45	274 ± 28	282 ± 24

Two-sided t-test was used for comparison between the control and knockout (KO) groups. For gsna/gsnb KO, Ventricular CV: *P=0.0109. For glipr2/glipr2l KO, Ventricular CV: **P=0.0085; Atrial CV: *P=0.0168; Ventricular Vmax: ****P<0.0001; Atrial Vmax: **P=0.0025; Ventricular APD80: **P=0.0090. For epn2 KO, Ventricular CV: *P=0.0201; Atrial Vmax: *P=0.0370; Ventricular APD80: *P=0.0185. For inf2 KO, Ventricular CV: ***P<0.0001. For nebl KO, Atrial CV: *P=0.0291; Ventricular APD80: *P=0.0484. CV=conduction velocity (ventricular CV: measured at the outer curvature, which has maximal magnitude); Vmax=maximum upstroke velocity (maximum dV/dt); APD_{80}=action potential duration at 80% repolarization.

Supplementary Table 6: Cardiac electrophysiology of zebrafish acute knockout II (Excel file).

Cardiac electrophysiology of zebrafish embryos (3 days post fertilization) with acute gene(s) knockout using multi-gRNA CRISPR/Cas9 (Excel file). Electrophysiological parameters characterized in CRISPANT knockout (KO) zebrafish and sibling control (WT) fish. $25^{th}/50^{th}/75^{th}$ percentiles of parameters acquired from atrial and ventricular chambers during sinus rhythm (SR) or pacing at 90/120 paces per minute (ppm). *p<0.05, **p<0.01, ***p<0.001 (exact p-values within the table) KO vs. WT sibling by 2-sided Mann-Whitney U test. APD20/50/80 = action potential duration at 20/50/80% repolarization. Triang = repolarization triangulation (time from APD20 to APD80). dVdt_20to80per = rate of action potential upstroke from 20 to 80% of action potential magnitude (%APAmp). Up50CVMag_OverallMedian = chamber global spatial median of conduction velocity magnitude perpendicular to activation wavefront (ventricular CV includes inner and outer curvatures, as well as proximal to the AV-canal). Up50CVMag_95Prct = chamber spatial 95th percentile of CV. Repol80Disp = spatial dispersion (standard deviation) of 80% repolarization time. APD80Disp = spatial dispersion of action potential duration at 80% repolarization.

Supplementary Table 7: Repolarization abnormality in adult nrap knockout zebrafish.

Electrocardiograms were collected from adult zebrafish with nrap knockout (KO) and their wildtype (WT) siblings. In some fish the magnitude of the T-wave of the signal-averaged ECG was of the same magnitude as the noise. This was disproportionately true of the nrap knockout fish.

	**Evident T-wave**	**Non-discernible from noise**
WT siblings	7	1
nrap KO	0	7

p=0.0014 by 2-sided Fisher's exact test.

Supplementary Table 8: Electrocardiogram parameters from adult knockout zebrafish (Excel file).

Characterization and comparison of electrocardiograms collected from adult knockout (KO) zebrafish and their wildtype (WT) siblings. $25^{th}/50^{th}/75^{th}$ percentiles of ECG parameters acquired during sinus rhythm under 0.016% tricaine anaesthesia. *p<0.05, **p<0.01 (exact p-values within the table) KO vs. WT sibling by 2-sided Mann-Whitney U test. QTc = heart rate corrected QT interval. For the nrap KO group, no repolarization (T-wave) dependent parameters are available. For pde4dip, all parameters that did not show a significant difference between group medians were subsequently tested for non-homogeneity of variance between groups by Conover squared-ranks test, †p<0.05 KO vs. WT sibling.

Supplementary Table 9: Table showing the data from protein interaction networks in Fig. 7 in table format (Excel file).

The table contains the data from the protein interaction networks in Fig. 7, the human ortholog gene names, a column indicating if an interactor was identified in our human heart proteome dataset (Fig. 7B), the mean mRNA expression values per cell type of each interactor extracted from Tucker et al. 2020 [1] (Fig. 7C), a column showing the maximum negative log10(p value) for the association of each interactors genetic loci with a time-resolved genetic influence on the ECG from ECGplotter [2] (Fig.7D) as well as quantitative proteomics data from human and mouse hearts from Linscheid et al. 2021 [3].

Supplementary Table 10: Comparison of protein expression in mouse and human hearts

The table shows Pearson correlation coefficients between human and mouse data for each chamber based on the proteomics data from Linscheid et al. 2021[3].

Comparison	Pearson's r
mouse LA vs human LA	0.8385
mouse RA vs human RA	0.9050
mouse LV vs human LV	0.8539
mouse LA vs mouse RA	0.9867
mouse LA vs mouse LV	0.7322
mouse LV vs mouse RV	0.9644
mouse RA vs mouse RV	0.7107
human LA vs human RA	0.9567
human LA vs human LV	0.7262
human RA vs human LV	0.5822

Supplementary Table 11: Percentage of interactors expressed in cardiomyocytes (Excel file).

The table is summarizing the percentage of interactors for each bait and of all baits combined that were found to be expressed in any of the cardiomyocyte subpopulations based on the snRNAseq data from Tucker et al.[1].

Supplementary Table 12: Summed mRNA expression of all interactors per cell type

The table shows the summed mRNA expression of all interactors for each cell type based on snRNAseq data from human hearts from Tucker et al. 2020. [1]

Cell type	Summed expression
04. Ventricular Cardiomyocyte I	295.85
03. Atrial Cardiomyocyte	226.25
11. Adipocyte	195.47
15. Ventricular Cardiomyocyte III	191.43
06. Ventricular Cardiomyocyte II	169.68
02. Fibroblast II	169.15
10. Endothelium II	161.54
13. Vascular Smooth Muscle	155.03
16. Neuronal	137.73
14. Fibroblast III	135.38
08. Macrophage	124.36
05. Cytoplasmic Cardiomyocyte I	114.93
12. Cytoplasmic Cardiomyocyte II	112.99
01. Fibroblast I	111.69
09. Endothelium I	103.01
07. Pericyte	94.54
17. Lymphocyte	71.27

Supplementary Table 13: Similarity of mRNA expression of interactors between cell types

The table shows Pearson correlation coefficients RNA expression of different cell populations and subpopulations based on snRNAseq data from human hearts from Tucker et al. 2020. [1]

Comparison	Pearson's r
Ventricular CM I vs. Ventricular CM II	0.9657
Ventricular CM I vs. Ventricular CM III	0.9472
Ventricular CM I vs. Atrial CM	0.9121
Ventricular CM II vs. Atrial CM	0.9052
Ventricular CM III vs. Atrial CM	0.9222
Ventricular CMI vs. Adipocyte	0.5435
Ventricular CM I vs. Macrophages	0.5369
Ventricular CM I vs. Fibroblast I	0.3079

Supplementary Figures

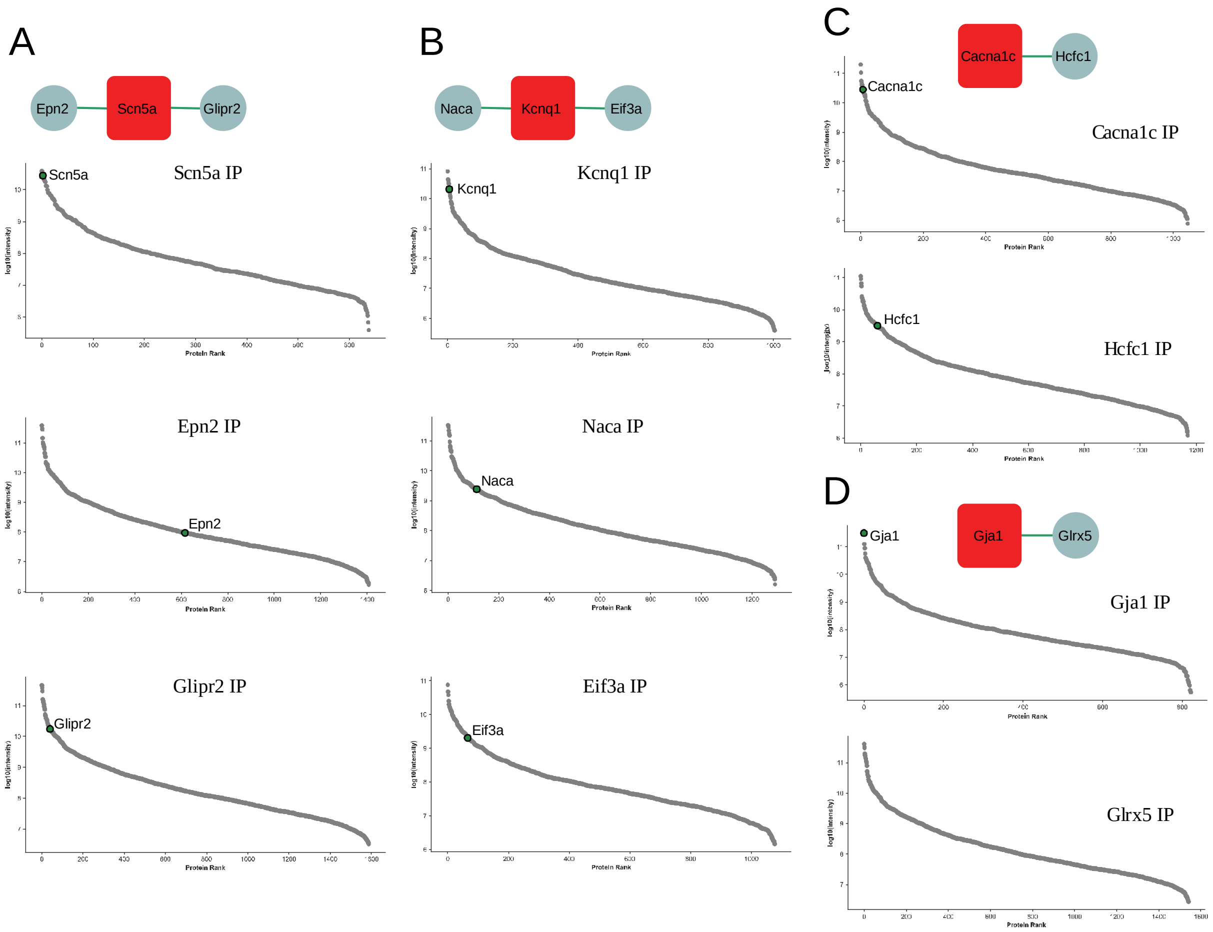

Supplementary Figure 1: Evaluating antibody specificities for reverse immunoprecipitations for a set of novel protein interactors. Immunoprecipitations were performed from murine cardiac tissue and precipitated proteins were evaluated by mass spectrometry. Protein rank plots show all proteins identified in each IP sorted by abundance in descending order with protein intensities plotted against protein ranks. In each panel it is indicated which ion channel the novel interactor was identified to be in interaction network with. In each panel, a prot ein rank plot is shown for the ion channel immunoprecipitations to illustrate results from this type of experiments when an antibody is deemed suitable for immunoprecipitations. A) Antibodies against the novel Scna5a interactors Epn2 and Glipr2 were evaluated. Epn2 was ranked protein number 617 out of 1408 (617/1408) and hence the antibody was not deemed suitable for immunoprecipitation. Glipr2 was ranked 41/1588. B) For Kcnq1, we tested antibodies against the two interactors Naca and Eif3a, that were ranked (114/1292) and (67/1078) respectively. C) For Cacna1c, we evaluated antibodies for its interactor Hcfc1 (61/1171). D) For Gja1, we tested an antibody against its interactor Glrx5. Glrx5 was not identified amongst the 1542 proteins in the IP. Antibody specificity for the six novel interactors was not high enough to pursue reverse immunoprecipitations.

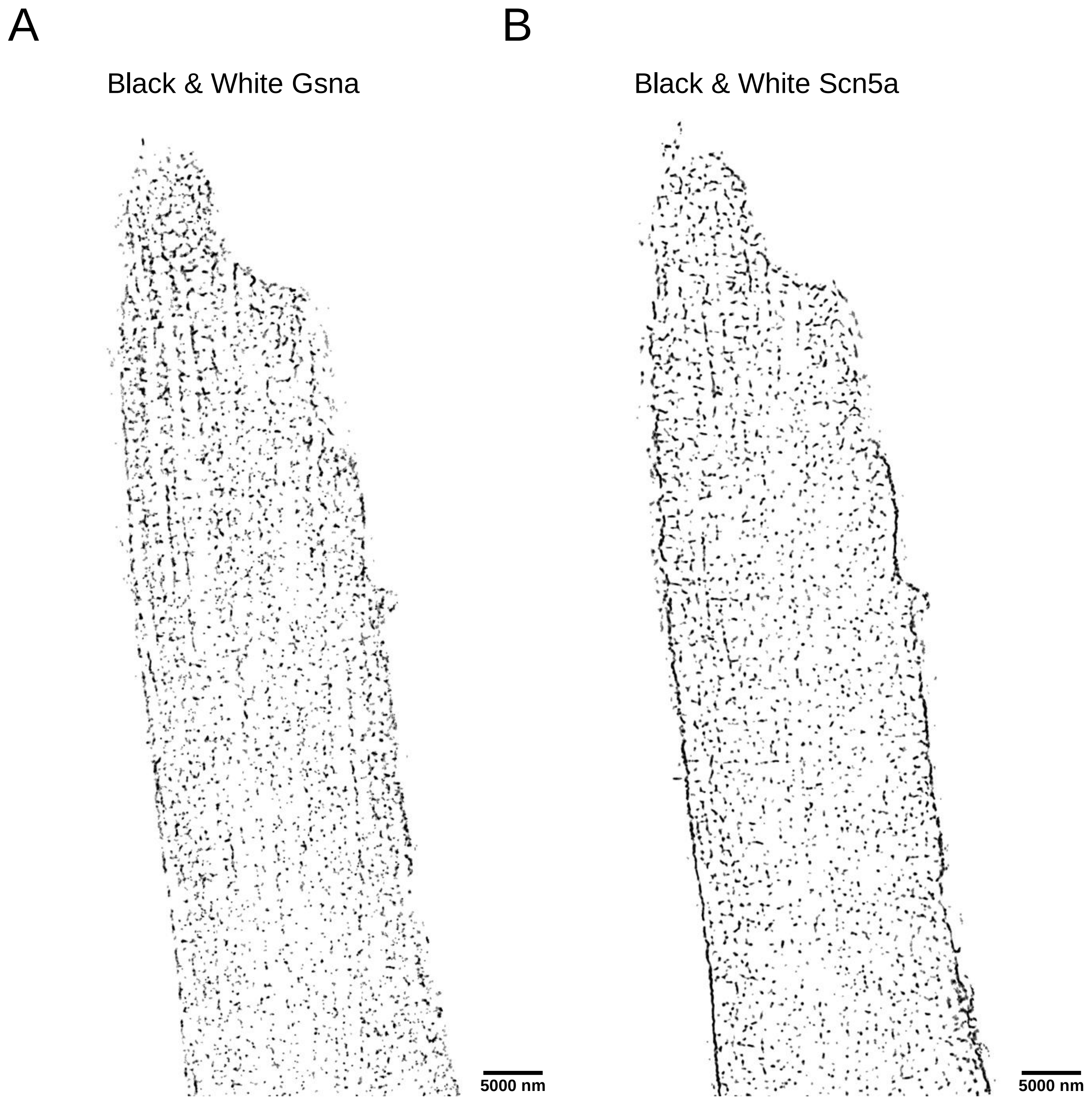

Supplementary Figure 2: Representative STORM images in black and white in murine cardiomyocytes. Representative STORM images of A) Gsn (Scn5a interactor), B) Scn5a. Scale bar included in the image. 15 cells examined over 3 mice in independent experiments with similar results.

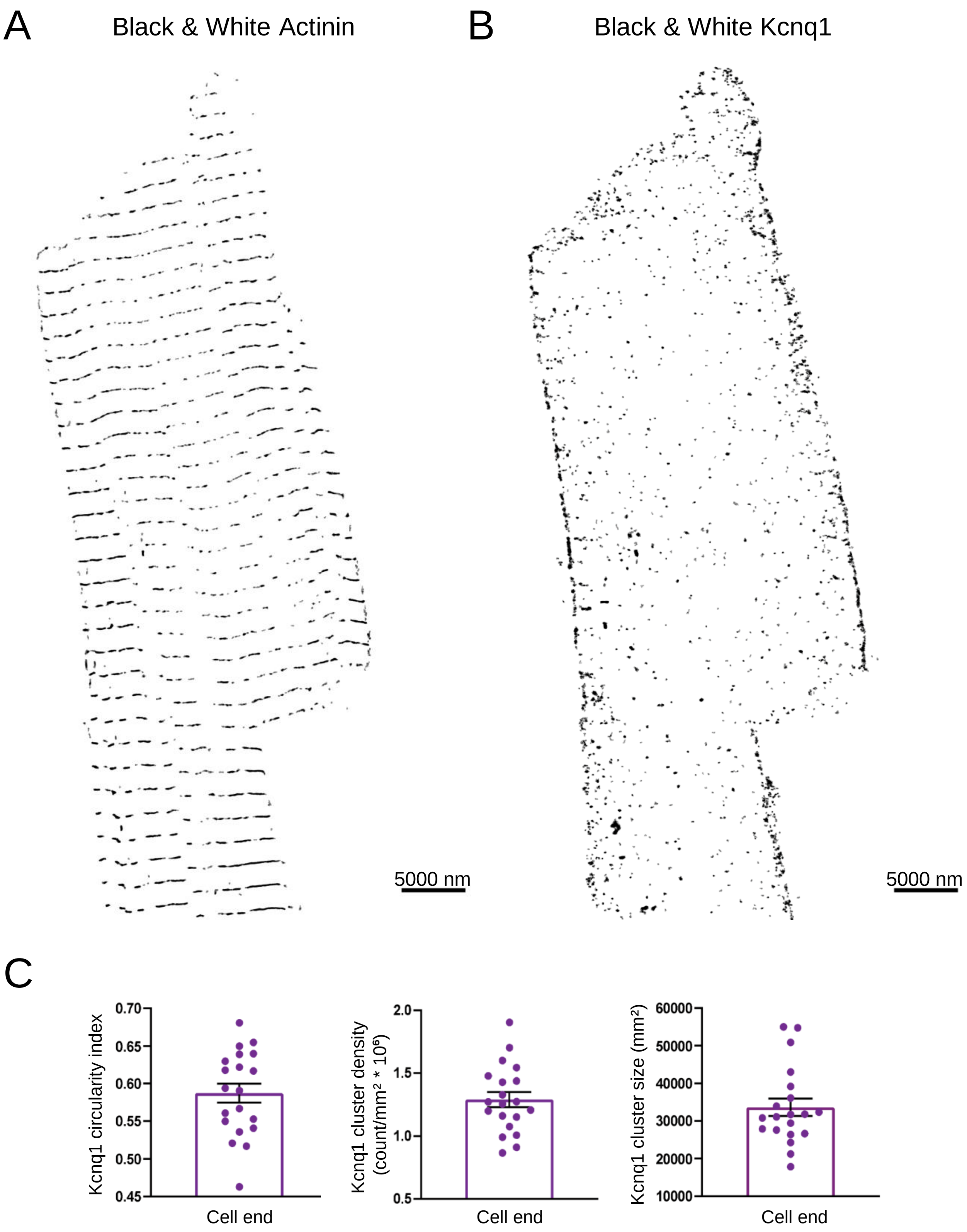

Supplementary Figure 3: STORM images in black and white from murine cardiomyocytes. Representative STORM images of A) α-actinin and B) Kcnq1 in murine cardiomyocytes. C) Kcnq1 cluster properties. The graphs show circularity, cluster density and cluster average size. 20 cells were examined over 3 mice in independent experiments. Data are presented as mean values +/- SEM.

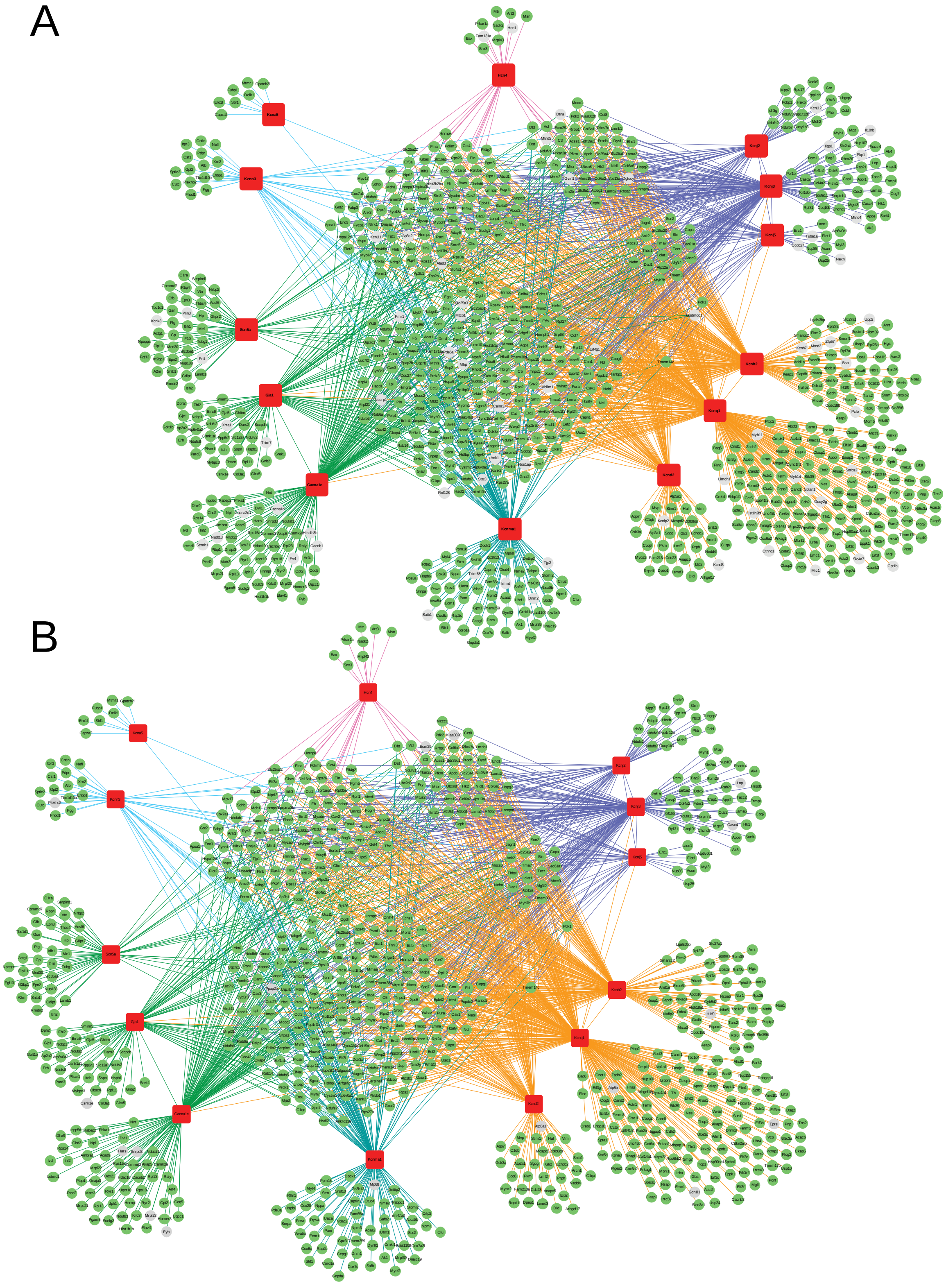

Supplementary Figure 4: ***Cardiac ion channel interaction network data intersection.*** Networks of all the proteins identified in this study (circles) to be significant interactors of the bait proteins (squares). *A) Intersection with human heart proteomics data.* The nodes in green are the ones found in our human cardiac proteome atlas (approximately 92% of all significant proteins), the nodes in grey were not found and are filtered out. B) *Intersection with human heart single-cell RNA sequencing data from Tucker et al.* [1]. Green circles indicate that an interactor was found to be expressed in at least one human cardiomyocyte subpopulation (98%), grey circles indicate that there was no evidence of expression in human cardiomyocytes and are filtered out.

Supplementary References

1 Tucker, N. R. *et al.* Transcriptional and Cellular Diversity of the Human Heart. *Circulation* **142**, 466-482, doi:10.1161/CIRCULATIONAHA.119.045401 (2020).

2 Verweij, N. *et al.* The Genetic Makeup of the Electrocardiogram. *Cell Syst* **11**, 229-238 e225, doi:10.1016/j.cels.2020.08.005 (2020).

3 Linscheid, N. *et al.* Quantitative proteome comparison of human hearts with those of model organisms. *PLoS Biol* **19**, e3001144, doi:10.1371/journal.pbio.3001144 (2021).
