# Supplementary material for: Outlining cardiac ion channel protein interactors and their signature in the human electrocardiogram
Source: Nat Cardiovasc Res. 2023 Jul 13;2(7):673–92. doi: 10.1038/s44161-023-00294-y (PMC11041666; doi:10.1038/s44161-023-00294-y)
Supplement: Supplementary file 2 — Reporting Summary [file 44161_2023_294_MOESM2_ESM.pdf]

nature portfolio

Corresponding author(s): Alicia Lundby

Last updated by author(s): May 18, 2023

Reporting Summary

Nature Portfolio wishes to improve the reproducibility of the work that we publish. This form provides structure for consistency and transparency in reporting. For further information on Nature Portfolio policies, see our Editorial Policies and the Editorial Policy Checklist.

Statistics

For all statistical analyses, confirm that the following items are present in the figure legend, table legend, main text, or Methods section.

n/a	Confirmed	
☐	☒	The exact sample size (*n*) for each experimental group/condition, given as a discrete number and unit of measurement
☐	☒	A statement on whether measurements were taken from distinct samples or whether the same sample was measured repeatedly
☐	☒	The statistical test(s) used AND whether they are one- or two-sided<br>*Only common tests should be described solely by name; describe more complex techniques in the Methods section.*
☐	☒	A description of all covariates tested
☐	☒	A description of any assumptions or corrections, such as tests of normality and adjustment for multiple comparisons
☐	☒	A full description of the statistical parameters including central tendency (e.g. means) or other basic estimates (e.g. regression coefficient) AND variation (e.g. standard deviation) or associated estimates of uncertainty (e.g. confidence intervals)
☐	☒	For null hypothesis testing, the test statistic (e.g. *F*, *t*, *r*) with confidence intervals, effect sizes, degrees of freedom and *P* value noted<br>*Give P values as exact values whenever suitable.*
☒	☐	For Bayesian analysis, information on the choice of priors and Markov chain Monte Carlo settings
☒	☐	For hierarchical and complex designs, identification of the appropriate level for tests and full reporting of outcomes
☐	☒	Estimates of effect sizes (e.g. Cohen's *d*, Pearson's *r*), indicating how they were calculated

Our web collection on statistics for biologists contains articles on many of the points above.

Software and code

Policy information about availability of computer code

Data collection	MaxQuant 1.6.2.3<br>Perseus 1.6.2.1<br>MATLAB R2019a<br>ImageJ 1.53a<br>Mahotas 1.2
Data analysis	R 4.0.3<br>LIMMA 3.36.0<br>Cytoscape 3.8.0<br><br>The R script for ECG plotter analysis is provided in our GitHub repository https://github.com/CardiacProteomics/ion-channel-interactomes.<br><br>Optical mapping analysis algorithms and Python script for STORM imaging have been previously described and the references are mentioned in the manuscript.

For manuscripts utilizing custom algorithms or software that are central to the research but not yet described in published literature, software must be made available to editors and reviewers. We strongly encourage code deposition in a community repository (e.g. GitHub). See the Nature Portfolio guidelines for submitting code & software for further information.

March 2021

Data

Policy information about availability of data

All manuscripts must include a data availability statement. This statement should provide the following information, where applicable:

- Accession codes, unique identifiers, or web links for publicly available datasets
- A description of any restrictions on data availability
- For clinical datasets or third party data, please ensure that the statement adheres to our policy

The mass spectrometry proteomics data have been deposited to the ProteomeXchange Consortium via the PRIDE partner repository with the dataset identifier PXD028021 and project name 'Cardiac ion channel interactomes'.

Mouse protein sequence database was downloaded from https://www.uniprot.org/, reviewed sequences only.

Human-mouse ortholog data was downloaded from Esembl BioMart: https://www.ensembl.org/info/data/biomart

Previously known interactors were extracted from:
- BioPlex: https://bioplex.hms.harvard.edu/interactions.php (BioPlex 3.0 Interactions (293T Cells))
- STRING: https://string-db.org/
- InWeb: https://doi.org/10.1038/nmeth.4083

snRNAseq data set was downloaded from Single Cell Portal (data set number SCP498): https://singlecell.broadinstitute.org/single_cell

Human ECG GWAS data downloaded from https://www.ecgenetics.org/.

Field-specific reporting

Please select the one below that is the best fit for your research. If you are not sure, read the appropriate sections before making your selection.

☒ Life sciences ☐ Behavioural & social sciences ☐ Ecological, evolutionary & environmental sciences

For a reference copy of the document with all sections, see nature.com/documents/nr-reporting-summary-flat.pdf

Life sciences study design

All studies must disclose on these points even when the disclosure is negative.

Sample size	No sample size calculation was performed. The sample size of four replicate immunoprecipitations per condition for mass spectrometry analysis were based on our previously published (PMID: 24952909 and PMID: 31315456) as well as unpublished data that defined the adequate number of samples to consistently identify channel interaction networks.<br>A minimum n of 9 zebrafish embryos was required for optical mapping studies, based on power calculations for effect size (Cohen's d) of 1.5 at p=0.05.
Data exclusions	The antibodies that weren't specific enough (i.e. didn't pull down the bait protein with high specificity) in the immunoprecipitation experiments were excluded from the dataset, as shown in Supplementary Figure S4.<br>For the zebrafish experiments only those isolated embryonic hearts that displayed evident characteristics of damage during microdissection (i.e. missing anatomy, substantial injury current) were excluded from data collection and analysis.
Replication	Gene knockout zebrafish data is pooled data from two independent replicates from different days. For three of the genes studied (nebl, nrap, and epn2), characterization of gene knockout zebrafish was replicated between investigational groups using independently designed CRISPR guide RNAs for the same gene target, recapitulating the knockout phenotypes.
Randomization	A clutch of fertilized zebrafish eggs at the single cell stage was agitated and swirled in a 10cm petri dish for at least 5 seconds by random movements to ensure adequate mixing before being divided at random into two groups of approximately equal number; these two groups are then immediately assigned at random to treatment or control.
Blinding	Mouse experiments:<br>Operators could not be blinded for identification of cells that received AAV infection because expression of GFP revealed the identity of the cells infected and therefore the cells suitable for patch clamp. A single operator conducted all experiments and therefore identity of the construct was revealed to the operator. However, all analysis was automated through operator independent patch clamp analysis software, and therefore no subject to a possible bias introduced by the operator.<br><br>Zebrafish experiments:<br>Zebrafish embryos develop rapidly but isolated hearts degrade with time, as such, hearts from treatment and control groups are isolated on demand and interleaved for successive imaging to minimize the potential for developmentally-associated differences between groups; this process effectively negates any blinding of individuals to group membership during optical mapping data acquisition. Extraction of electrophysiological parameters from these recordings is performed by automated and objective computer algorithms, and as such blinding of

this analysis was not relevant. For ECG recordings from adult zebrafish, the author who acquired and analyzed the ECGs was blinded to genotype.

Reporting for specific materials, systems and methods

We require information from authors about some types of materials, experimental systems and methods used in many studies. Here, indicate whether each material, system or method listed is relevant to your study. If you are not sure if a list item applies to your research, read the appropriate section before selecting a response.

Materials & experimental systems

n/a	Involved in the study
☐	☒ Antibodies
☒	☐ Eukaryotic cell lines
☒	☐ Palaeontology and archaeology
☐	☒ Animals and other organisms
☒	☐ Human research participants
☒	☐ Clinical data
☒	☐ Dual use research of concern

Methods

n/a	Involved in the study
☒	☐ ChIP-seq
☒	☐ Flow cytometry
☒	☐ MRI-based neuroimaging

Antibodies

Antibodies used

Antibodies used for immunoprecipitations:

Sr. No.	Antibody cat #	Description
1	APC022 (Alomone)	Anti KV7.1
2	APC062 (Alomone)	Anti KV11.1
3	ACC-003 (Alomone)	Anti-CaV1.2
4	ASC-005 (Alomone)	Anti-NaV1.5
5	APC-004 (Alomone)	Anti-KV1.5
6	APC-023 (Alomone)	Anti-KV4.2
7	APC-052 (Alomone)	Anti HCN4
8	APC-026 (Alomone)	Anti-Kir2.1
9	APC-005 (Alomone)	Anti-Kir3.1 (GIRK1)
10	APC-027 (Alomone)	Anti-Kir3.4 (GIRK4)
11	APC-021 (Alomone)	Anti-KCa1.1 (1097-1196)
12	ACC-201 (Alomone)	Anti Cx43
13	APC-025 (Alomone)	Anti-Kcnn3
14	ab37415 (Abcam)	Control IgG Rabbit
15	A303-428A (Bethyl labs)	Anti-Inf2

The antibodies were used in a 1:1000 ratio for immunoblots and 2 μg antibody was used for immunoprecipitation experiments per replicate.

Antibodies used for STORM imaging:
-mouse Anti-Connexin 43 clone 4E6.2 (1:50, Sigma-Aldrich catalog number # MAB3067, Lot: 3138211)
-rabbit polyclonal Scn5a (1:50, Sigma catalog number #S0819, Lot: SLBW8952)
-rabbit polyclonal Kcnq1 (1:50, Alomone catalog number #APC-022)
-rabbit polyclonal Kcnq1 (1:50, Alomone catalog number #APC-168)
-rabbit monoclonal anti-Gelsolin clone EPR1941Y conjugated to Alexa Fluor 647 (1:100, Abcam catalog number #ab75832)
-mouse anti-αActinin clone EA-53 conjugated to Alexa Fluor 488 (1:300, Sigma-Aldrich catalog number # A7811 Lot:0000141496)
-Alexa Fluor goat anti-rabbit 568 (1:10000, Invitrogen, catalog number #A11011, Lot: 1778925),
-Alexa Fluor goat anti-rabbit 647 (1:10000, Invitrogen, catalog number #A21244, Lot: 1834794)
-Alexa Fluor goat anti-mouse 488 (1:10000, Invitrogen, catalog number #A11001, Lot: 2220848).

Validation

Please find the validation text for the antibodies used from the manufacturer's website. The website itself has been mentioned in parenthesis, should more information be required.

1. Anti-KCNQ1 Antibody (#APC-022) is a highly specific antibody directed against an epitope of the human protein. The antibody can be used in western blot, immunoprecipitaion, immunohistochemistry and immunocytochemistry applications. It has been designed to recognize KV7.1 channel from rat, human, and mouse samples.(https://www.alomone.com/p/anti-kv7-1-kcnq1/APC-022)

2. Anti-KCNH2 (HERG) Antibody (#APC-062) can be used in western blot, immunoprecipitation, immunohistochemical and immunocytochemical applications. It has been designed to recognize intracellular epitope of KV11.1 from human, rat, and mouse samples.(https://www.alomone.com/p/anti-kv11-1-herg/APC-062)

3. Anti-CaV1.2 (CACNA1C) Antibody (#ACC-003) is a highly specific antibody directed against an epitope of the rat protein. The antibody can be used in western blot, immunoprecipitation, immunohistochemistry, immunocytochemistry, and indirect flow cytometry applications. It has been designed to recognize CaV1.2 from mouse, rat, and human samples.(https://www.alomone.com/p/anti-cav1-2-antibody/ACC-003)

4. Anti-NaV1.5 (SCN5A) (493-511) Antibody (#ASC-005) is a highly specific antibody directed against an epitope of the rat protein. The antibody can be used in western blot, immunoprecipitation, immunohistochemistry, and immunocytochemistry applications. It has been designed to recognize NaV1.5 sodium channel from rat, human, and mouse samples.(https://www.alomone.com/p/anti-nav1-5/ASC-005)

5. Anti-KV1.5 (KCNA5) Antibody (#APC-004) is a highly specific antibody directed against an epitope of the mouse protein. The antibody can be used in western blot, immunohistochemistry, immunocytochemistry, and immunoprecipitation applications. It has been designed to recognize KV1.5 from human, rat, and mouse samples.(https://www.alomone.com/p/anti-kv1-5/APC-004)

6. Anti-KV4.2 Antibody (#APC-023) is a highly specific antibody directed against an epitope of the rat protein. The antibody can be used in western blot, immunoprecipitation, immunocytochemistry, and immunohistochemistry applications. It has been designed to recognize KV4.2 from human, rat, and mouse samples.(https://www.alomone.com/p/anti-kv4-2/APC-023)

7. Anti-HCN4 Antibody (#APC-052) is a highly specific antibody directed against an epitope of the human protein. The antibody can be used in western blot, immunoprecipitation, immunocytochemistry, and immunohistochemistry applications. It has been designed to recognize HCN4 from human, rat, and mouse samples.(https://www.alomone.com/p/anti-hcn4-2/APC-052)

8. Anti-Kir2.1/KCNJ2 Antibody (#APC-026) can be used in western blot, immunoprecipitation, immunocytochemistry, immunohistochemistry, and flow cytometry applications. It has been designed to recognize Kir2.1 from human, rat, and mouse samples.(https://www.alomone.com/p/anti-kir2-1/APC-026)

9. Anti-GIRK1 (Kir3.1) Antibody (#APC-005) is a highly specific antibody directed against an epitope of the mouse protein. The antibody can be used in western blot, immunprecipitation, immunocytochemistry, and immunohistochemistry applications. It has been designed to recognize Kir3.1 from human, rat, and mouse samples.(https://www.alomone.com/p/anti-kir3-1-girk1/APC-005)

10. Anti-KCNJ5 (Kir3.4) Antibody (#APC-027) can be used in western blot and immunohistochemistry applications. It has been designed to recognize Kir3.4 from human, rat and mouse samples.(https://www.alomone.com/p/anti-kir3-4-girk4/APC-027)

11. Anti-KCNMA1 (KCa1.1) (1097-1196) Antibody (#APC-021) is a highly specific antibody directed against an epitope of the mouse protein. The antibody can be used in western blot, immunoprecipitation, immunohistochemistry, and immunocytochemistry applications. It has been designed to recognize KCNMA1 from human, mouse, and rat samples.(https://www.alomone.com/p/anti-kca1-1-1097-1196/APC-021)

12. Anti-Connexin-43 Antibody (#ACC-201) can be used in western blot, immunohistochemistry and immunocytochemistry applications. It has been designed to recognize Cx43 from human, rat and mouse samples.(https://www.alomone.com/p/anti-connexin-43/ACC-201)

13. Anti-KCNN3 (KCa2.3, SK3) (N-term) Antibody (#APC-025) is a highly specific antibody directed against an intracellular epitope at the N-terminus of the human KCNN3 channel. The antibody can be used in western blot, immunocytochemistry, and immunohistochemistry applications. It has been designed to recognize KCNN3 from human, rat, and mouse samples.(https://www.alomone.com/p/anti-kca2-3-sk3-n-term/APC-025)

14. ab37415 This antibody has been selected to be an isotype control as it has no known specificity. Please note that the rabbit IgG Fc region may bind nonspecifically to human tissue. Rabbit IgG is an isotype control used to estimate the non-specific binding of target primary antibodies due to Fc binding or other protein-protein interactions.(https://www.abcam.com/rabbit-igg-polyclonal-isotype-control-ab37415.html)

15. INF2 Antibody, A303-428A
Rabbit anti-INF2 Antibody, Affinity Purified
Reactivity Human
Applications WB, IP
Host Rabbit
Antibody Type Polyclonal
Conjugate Unconjugated
Format Whole IgG
Immunogen between 950 and 1000 (https://www.bethyl.com/product/A303-428A/INF2+Antibody)

16. ab75832: Produced recombinantly (animal-free) for high batch-to-batch consistency and long term security of supply
Rabbit monoclonal [EPR1941Y] to Gelsolin plasma
Suitable for: WB, IP, IHC-P
Reacts with: Mouse, Human (https://www.abcam.com/gelsolin-plasma-antibody-epr1941y-ab75832.html)

17. A7811: Quality Level: 200
biological source:mouse
antibody form: ascites fluid
antibody product type: primary antibodies
clone: EA-53, monoclonal
mol wt: antigen 100 kDa
species reactivity: fish, snake, frog, goat, hamster, pig, canine, mouse, feline, chicken, lizard, bovine, human, sheep, rat, rabbit
application(s): immunohistochemistry (formalin-fixed, paraffin-embedded sections): 1:800 using human skeletal and cardiac muscle
isotype: IgG1 (https://www.sigmaaldrich.com/US/en/product/sigma/a7811)

18. S0819: biological source: rabbit
Quality Level: 100
antibody form: affinity isolated antibody

antibody product type: primary antibodies
clone: polyclonal
species reactivity: rat
application(s): western blot: 1:200 using rat heart membranes
conjugate: unconjugated (https://www.sigmaaldrich.com/US/en/product/sigma/s0819)

19. MAB3067: Anti-Connexin 43 Antibody, clone 4E6.2 detects level of Connexin 43 & has been published & validated for use in ELISA, IC, IH & WB.(https://www.emdmillipore.com/US/en/product/Anti-Connexin-43-Antibody-clone-4E6.2,MM_NF-MAB3067?ReferrerURL=https%3A%2F%2Fwww.google.com%2F)

Animals and other organisms

Policy information about studies involving animals; ARRIVE guidelines recommended for reporting animal research

Laboratory animals	1. Immunoprecipitation experiments: Male mouse C57BL/6JRj (Janvier Labs), 8 weeks old. Mice were housed in individually ventilated cage-systems with 8-10 ACH (air changes per hour), temperature: 22C (+/- 2C), humidity: 55% (+/-10%) and standard 12:12h light:dark cycle. Food and water were provided ad libitum.<br>2. Zebrafish studies: Wild-type (WT) AB/Tuebingen (AB/Tu) zebrafish between the ages of 5-16 months were mated and the resultant embryos (3-5 dpf) or adult (3-4 months) fish (age-matched clutch-mates within the context of each individual study), both male and female, were used for the studies.<br>3. Cardiomyocyte dissociation experiments for STORM imaging: Male and female C57BL/6 N, MHC Haplotype: H2b, Strain Code: 027, (Charles River Laboratories), 3-4 months old, Temperature (21-23°C) and relative humidity (30-70%) were maintained according to standard protocols put in place by the Division of Comparative Medicine at NYU Grossman School of Medicine. Lighting was provided via an automatic timer with 12 hours light-dark cycle.
Wild animals	The study did not involve wild animals
Field-collected samples	The study did not involve samples collected from the field
Ethics oversight	Immunoprecipitation experiments:All animal experiments using mouse were performed according to the European Union legislation for protection of animals used for scientific experiments. All animal experiments using zebrafish were performed according to the European Union legislation for protection of animals used for scientific experiments and was approved by the Danish National Animal Experiments Inspectorate (license 2021-15-0201-00811) or are approved by the Institutional Animal Care and Use Committee at Brigham and Women's Hospital and Harvard Medical School. Cardiomyocyte dissociation experiments for STORM imaging: Procedures conformed with the Guide for Care and Use of Laboratory Animals of the National Institutes of Health and were approved by the New York University Institutional Animal Care and Use Committee under protocol number 160726-03.

Note that full information on the approval of the study protocol must also be provided in the manuscript.
