# Supplementary material for: Outlining cardiac ion channel protein interactors and their signature in the human electrocardiogram
Source: Nat Cardiovasc Res. 2023 Jul 13;2(7):673–92. doi: 10.1038/s44161-023-00294-y (PMC11041666; doi:10.1038/s44161-023-00294-y)
Supplement: Supplementary file 11 — Unprocessed western blots. [file 44161_2023_294_MOESM11_ESM.pdf]

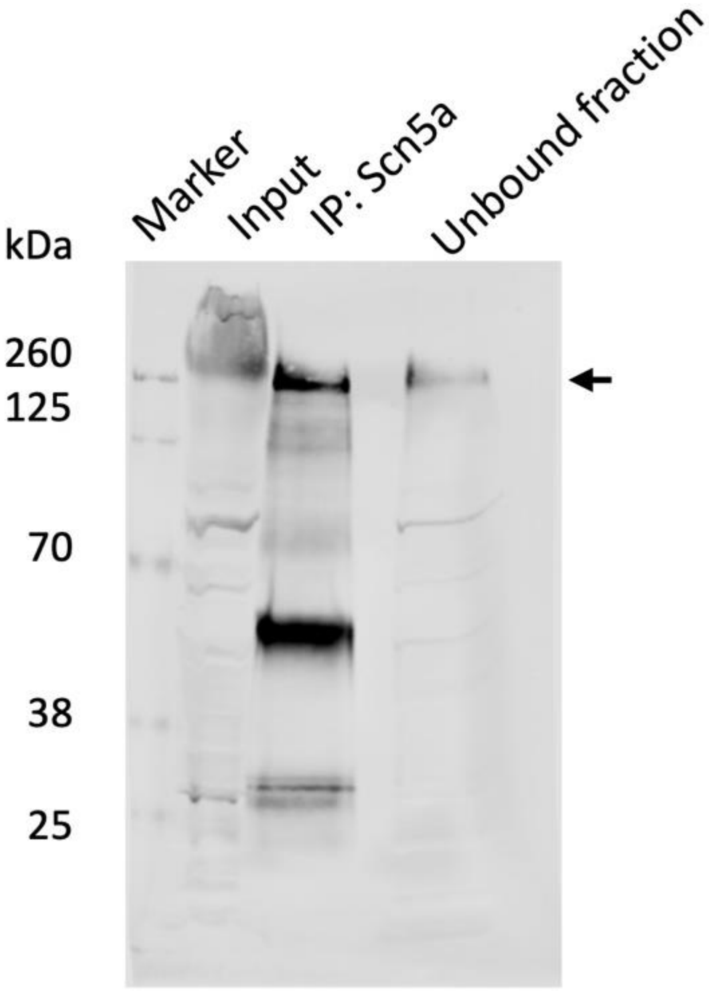

Source data for Extended Data Fig. 1A

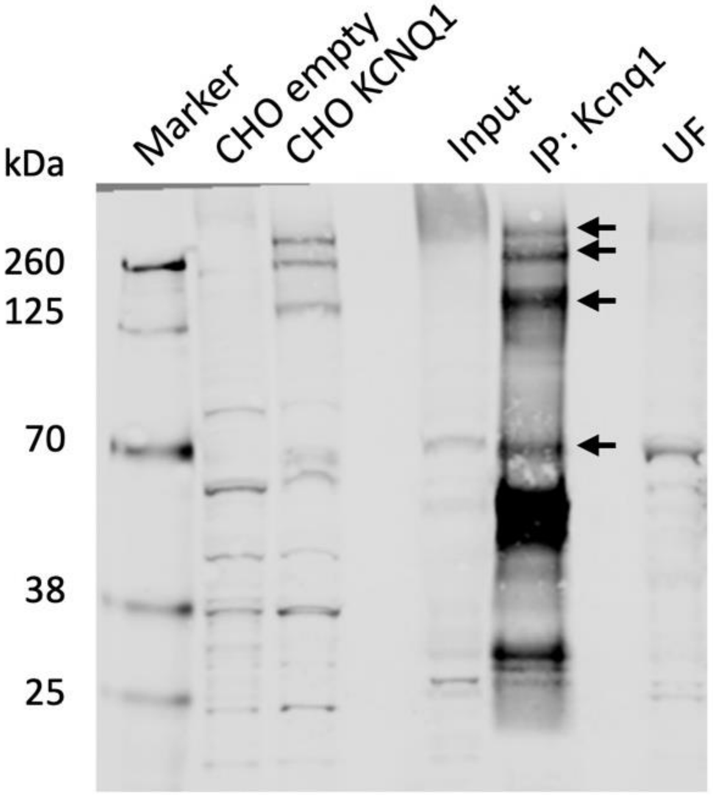

Source data for Extended Data Fig. 1B

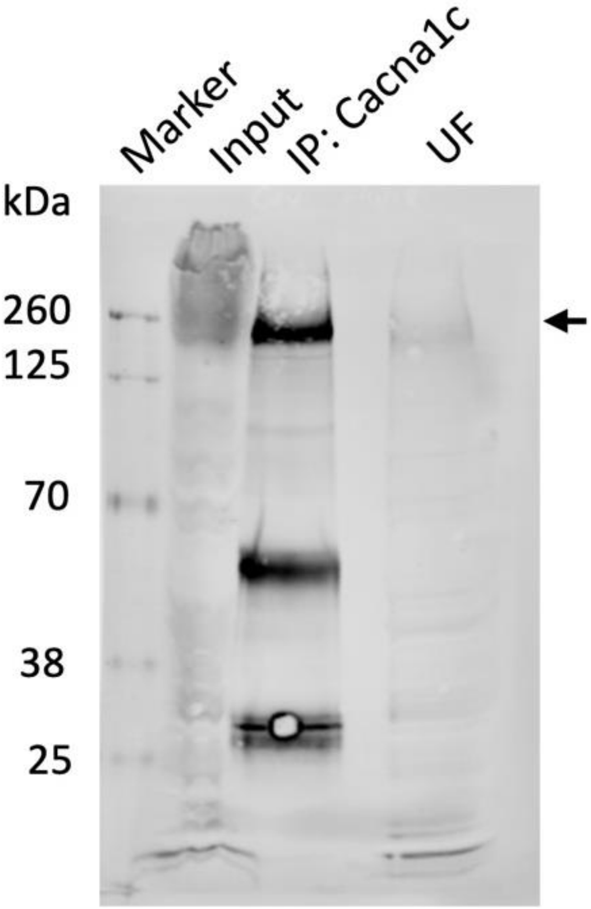

Source data for Extended Data Fig. 1C

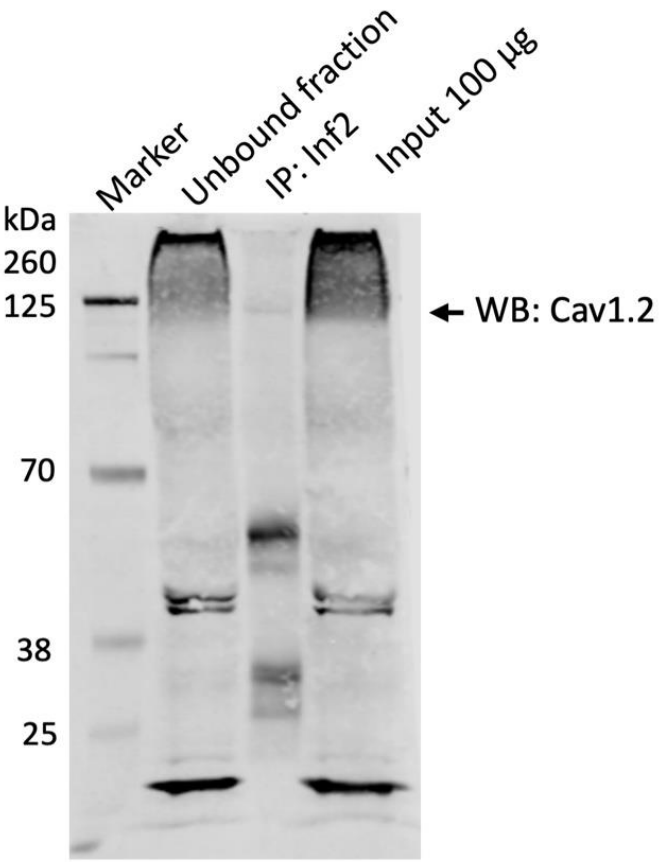

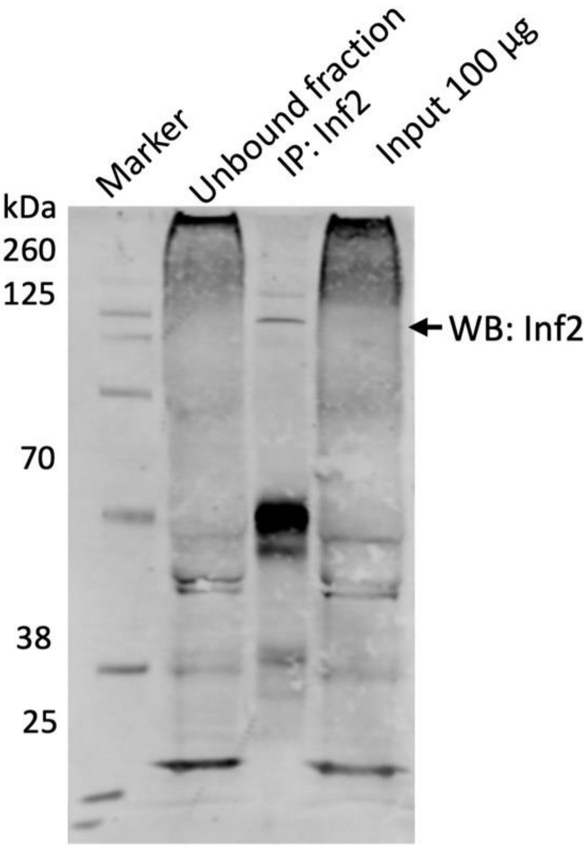

Source data for Extended Data Fig. 1D
